# Phytochemical Study and In Vitro Screening Focusing on the Anti-Aging Features of Various Plants of the Greek Flora

**DOI:** 10.3390/antiox10081206

**Published:** 2021-07-28

**Authors:** Aimilia D. Sklirou, Maria T. Angelopoulou, Aikaterini Argyropoulou, Eliza Chaita, Vasiliki Ioanna Boka, Christina Cheimonidi, Katerina Niforou, Eleni Mavrogonatou, Harris Pratsinis, Eleftherios Kalpoutzakis, Nektarios Aligiannis, Dimitris Kletsas, Ioannis P. Trougakos, Alexios Leandros Skaltsounis

**Affiliations:** 1 Department of Cell Biology and Biophysics, Faculty of Biology, National and Kapodistrian University of Athens, 15784 Athens, Greece; asklirou@biol.uoa.gr (A.D.S.); chrischeim@biol.uoa.gr (C.C.); kniforou@biol.uoa.gr (K.N.); 2 Laboratory of Cell Proliferation and Ageing, Institute of Biosciences and Applications, NCSR ‘‘Demokritos’’, 15310 Athens, Greece; mangelopoulou@bio.demokritos.gr (M.T.A.); elmavro@bio.demokritos.gr (E.M.); hprats@bio.demokritos.gr (H.P.); 3 Division of Pharmacognosy and Natural Products Chemistry, Department of Pharmacy, National and Kapodistrian University of Athens, Panepistimiopolis Zografou, 15771 Athens, Greece; katarg@pharm.uoa.gr (A.A.); elchaita@pharm.uoa.gr (E.C.); vana_b@otenet.gr (V.I.B.); elkalp@pharm.uoa.gr (E.K.); aligiannis@pharm.uoa.gr (N.A.); 4 Institute of Organic and Analytical Chemistry (ICOA), UMR 7311, Université d’Orléans, rue de Chartres, 45067 Orléans, France

**Keywords:** skin health, aging, plant extracts, natural products, phytochemistry, *Sideritis scardica*, *Rosa damascena*, senescence, photoprotection, proteasome

## Abstract

Skin health is heavily affected by ultraviolet irradiation from the sun. In addition, senile skin is characterized by major changes in the collagen, elastin and in the hyaluronan content. Natural products (NPs) have been shown to delay cellular senescence or in vivo aging by regulating age-related signaling pathways. Moreover, NPs are a preferable source of photoprotective agents and have been proven to be useful against the undesirable skin hyperpigmentation. Greek flora harvests great plant diversity with approximately 6000 plant species, as it has a wealth of NPs. Here, we report an extensive screening among hundreds of plant species. More than 440 plant species and subspecies were selected and evaluated. The extracts were screened for their antioxidant and anti-melanogenic properties, while the most promising were further subjected to various in vitro and cell-based assays related to skin aging. In parallel, their chemical profile was analyzed with High-Performance Thin-Layer Chromatography (HPTLC) and/or Ultra-Performance Liquid Chromatography High-Resolution Mass Spectrometry (UPLC-HRMS). A variety of extracts were identified that can be of great value for the cosmetic industry, since they combine antioxidant, photoprotective, anti-melanogenic and anti-aging properties. In particular, the methanolic extracts of *Sideritis scardica* and *Rosa damascena* could be worthy of further attention, since they showed interesting chemical profiles and promising properties against specific targets involved in skin aging.

## 1. Introduction

Aging is an inevitable process, which affects most living organisms and is defined as the time-dependent decline of biological functions and stress response capacity [1]. These events lead to the gradual accumulation of damaged biomolecules, which jeopardize cellular homeodynamics, increasing thus the risk for human diseases, including neurodegenerative disorders and cancer [2,3].

Organisms constantly encounter a variety of stressors, both endogenous (e.g., mitochondria-derived reactive oxygen species (ROS)) and exogenous (e.g., oxidants, heat shock, radiation, diet-derived reducing sugars or reactive aldehydes) [4]. It is generally accepted that oxidative stress, a situation in which ROS levels exceed the antioxidant capacity of cells, is a major risk factor in aging and most age-related diseases [5]. Notably, increased oxidative load can damage stochastically all biomolecules leading to extensive genome and proteome instability [6].

In addition, the ability to maintain a functional proteome, or proteostasis, declines during the aging process [7,8]. Loss of proteostasis has been recognized as a hallmark of aging, highlighting the great significance of proteome integrity in cellular functionality and survival [1,9]. Proteome stability is achieved by the multi-compartmental modular proteostasis network (PN). PN regulates protein synthesis, folding, transport, and degradation of damaged proteins and also prevents accumulation of protein aggregates [7,10]. Damaged proteins are degraded by the two main proteolytic systems of PN, namely the ubiquitin proteasome (UPP) and the autophagy-lysosome pathways [10,11]. Several reports suggest that activation of either proteasome activity or autophagic-lysosomal potential extends lifespan and delays the onset of age-related diseases [8,10].

Skin, one of the largest organs in the body and the main barrier between the organism and the environment, is subject to aging caused by both intrinsic and extrinsic factors. Senile skin is characterized mainly by alterations of the dermis, such as a diminished proliferative capacity of the cells with a concomitant loss of cells, and major changes in the architecture of the collagen and elastin networks, as well as, in the hyaluronan content, while the alterations in the epidermis are minor [12,13,14]. A major insult affecting skin health throughout our life is the ultraviolet (UV) irradiation from the sun, which directly causes oxidative stress and DNA damage in skin cells. This in turn triggers topical inflammatory reactions recruiting infiltrating neutrophils [15], and ultimately leads to premature skin aging (photoaging) and skin cancer [16]. Many of the deleterious results of UV are mediated by key enzymes produced by the skin cells or the infiltrating neutrophils, such as matrix metalloproteinases (MMPs), and especially MMP-1, -3, and -9, elastase and hyaluronidase [17,18].

Nowadays, it is evident that natural products (NPs) isolated from different sources of the biosphere (e.g., plants, marine organisms, microorganisms) could delay cellular senescence of normal human cells or in vivo aging of model organisms by regulating age-related signaling pathways [19,20,21]. NPs are also a preferable source of novel photoprotective agents, since they usually combine UV absorption with antioxidant, anti-inflammatory, and immunomodulatory properties [22]. Moreover, NPs have been proven to be useful for addressing another symptom of photoaging, i.e., undesirable skin hyperpigmentation, through inhibition of melanogenesis [23]. During a previous search among 600 taxonomically diverse Panamanian plant extracts, we have isolated a series of known polyphenols with antioxidant and photoprotective properties [24]. 

Greece, and especially the southern part of the country, harvests the most diverse flora in Mediterranean. It has great floristic wealth and is considered one of the global ecological “hot spots”. Due to the geographic position and the coexistence of three floral regions, Greek flora consists of approximately 6000 species of higher plants. Of these, ~13% of the plant taxa are endemic making Greece an area of high conservation priority [25]. Here, we report an extensive screening among hundreds plant species of the Greek flora in order to identify bioactive natural extracts against age-related proteome instability, and cellular senescence, as well as against more specific targets involved in skin aging, such as melanogenesis, phototoxicity and extracellular matrix (ECM) catabolism.

## 2. Materials and Methods

### 2.1. Plant Material

Dr. E. Kalpoutzakis collected and identified 450 samples of species and subspecies of Greek flora (wild and cultivated) from several areas of the Greek province. Details about the place of collection and the plant part are presented in Supplementary Table S1. Voucher specimens have been deposited at the Herbarium of the Department of Pharmacognosy and Natural Products Chemistry.

### 2.2. Extraction

Pulverized, air-dried plant parts were extracted with pressurized liquid extraction. For that purpose, a Dionex Accelerated solvent extraction (ASE) 300 System (Dionex, Sunnyvale, CA, USA) with 100 mL stainless steel vessels was used. Specifically, 20 g of the plants were placed into the tubular extraction cells. These were then placed into the carousel and the samples were extracted under specified conditions: temperature 70 °C, pressure 120 bar (preset by the instrument), pre-heat time 1 min, heat time 5 min, 2 extraction cycles of 5 min static time each, Flush volume 100% and Purge 120 s with nitrogen gas. Separate preparations with ethyl acetate and methanol solvents (analytical grade) were performed. The final extracts were collected in clear glass vials (250 mL). The solvents were evaporated to dryness under reduced pressure using a rotary evaporator (Buchi Rotavapor R-200, Buchi Labortechnik AG, Flawil, Switzerland) at 40 °C.

### 2.3. 2,2-Diphenyl-1-Picrylhydrazyl (DPPH) Radical Scavenging Activity

The ability of the extracts to scavenge DPPH free radicals was determined according to a previously described method [26], with slight modifications for application in a 96-well plate reader. A stock DPPH solution (0.314 mM) was prepared in absolute ethanol, vortexed and kept in the dark at room temperature until its use. The plant extracts were diluted in dimethyl sulfoxide (DMSO) at final concentration of 0.20 mg/mL. In a microwell plate, 190 μL of the DPPH solution and 10 μL of gallic acid or samples were added. The changes in color were read at 517 nm after 30 min of incubation in the dark at room temperature using the reader Infinite 200 PRO series (Tecan GmbH, Crailsheim, Germany). Gallic acid (purity > 98%) was used as a positive control. Negative control was composed of 190 µL of DPPH solution and 10 µL of DMSO. For the blanks 190 µL of EtOH and 10 µL of sample were used. Experiments were performed in triplicate for each sample. All materials were purchased from Merck KGaA (Darmstadt, Germany). The percentage of radical scavenging activity of the tested extracts was determined as follows:

{1 − [(A_sample_ − A_blank_)/A_control_]} × 100, where A_control_ is the absorbance of the negative control, A_sample_ is the absorbance after the reaction of samples with DPPH, and A_blank_ is the absorbance of samples without DPPH. The IC_50_ values were determined for the most promising extracts (% inhibition of DPPH > 90% at 0.20 mg/mL), at concentrations ranging from 0.20 mg/mL to 0.0125 mg/mL.

### 2.4. Tyrosinase Inhibition

The ability of the samples to inhibit the oxidation of L-3,4-dihydroxyphenylalanine (L-DOPA) to dopaquinone and subsequently to dopachrome was investigated employing a modification of an established protocol [27]. The extracts were dissolved in DMSO and diluted in the proper concentration (0.3 mg/mL) in phosphate buffer 1/15 M (NaH_2_PO_4_/Na_2_HPO_4_), pH 6.8. The final concentration of DMSO in the well never exceeded 3%. In 96-well plates, 80 μL of phosphate buffer 1/15 M, 40 μL of sample and 40 μL of mushroom tyrosinase (92 Units/mL) solutions in the same buffer were mixed. The content of each well was incubated for 10 min at 23 °C, before 40 μL of 2.5 mM L-DOPA in the same buffer were added. After incubating at 23 °C for 5 min, the absorbance at 475 nm of each well was measured, using the Infinite 200 PRO series plate reader (Tecan GmbH, Crailsheim, Germany). Blanks for every sample (*w*/*o* tyrosinase) were also performed, while Kojic acid was used as positive control. Measurements were made in triplicate. The percentage inhibition of tyrosinase activity was calculated by the following equation: [(A − B) − (C − D)]/(A − B) × 100, where A: Control (*w*/*o* sample), B: Blank (*w*/*o* sample, *w*/*o* tyrosinase), C: Sample, D: Blank sample (*w*/*o* tyrosinase). The IC_50_ values were determined for the most promising extracts (% inhibition of tyrosinase >40% at 0.30 mg/mL), at concentrations ranging from 0.30 mg/mL to 0.050 mg/mL.

### 2.5. Hyaluronidase Inhibition

The enzymatic assay for the inhibition of hyaluronidase was conducted as described by Algul et al. [28], with some modifications. The plant extracts were diluted to DMSO to a final concentration of 0.30 mg/mL. The inhibition activity was calculated inversely proportional of the production of N-acetyl-D-glucosamine. Values of 100 μL of acetate buffer (0.1 M NaCl, pH 3.5), 150 μL of tested sample and 50 μL of hyaluronidase solution 1% *w*/*v* (dissolved in acetate buffer) were added in eppendorfs. Afterwards, 100 μL of BSA solution 0.2% *w*/*v* (dissolved in ddH_2_O) was added in each eppendorf and incubated for 20 min at 37 °C. Then, 50 μL of hyaluronic acid solution 0.5% *w*/*v* (dissolved in ddH_2_O) was added and incubated for 60 min at 37 °C; 45 μL from each eppendorf was transferred into new eppendorfs containing 10 μL of sodium tetraborate solution 0.8 M (dissolved in ddH_2_O), and heated for 3 min at 100 °C and cooled down on ice. In each tube 300 μL of dimethylaminobenzaldehyde (DMAB) solution was added (10% *w*/*v* dissolved in 10 N HCl and then dissolved 10 times in acetic acid glacial) and incubated for 20 min at 37 °C. Finally, 200 μL from the last tube was transferred into a 96-well microplate and measured at 586 nm in the Infinite 200 PRO series plate reader (Tecan GmbH, Crailsheim, Germany). Tannic acid was used as positive control and DMSO instead of sample for negative control. Experiments were performed in triplicate.

The activity was calculated depending on the change in absorption using the following equation: Activity (%) = [A − (B − C)]/A ∗ 100,where A is the absorbance of the negative control, B is the absorbance of the tested sample and C is the absorbance of zero value. 

### 2.6. Elastase Inhibitory Activity

The elastase inhibitory activity was tested spectrophotometrically according to a previously described method [29], with slight modifications. The porcine pancreatic elastase type IV (PPE), a lyophilized powder at ≥4 units/mg protein, was used for this bioassay. Crude extracts were evaluated at the concentration of 0.3 µg/mL. N-succinyl-Ala-Ala-Ala-p-nitroanilide was used as a substrate and the release of p-nitroaniline was monitored. The reaction mixture initially contained 70 μL Trizma-base buffer (50 mM, pH 7.5), 10 μL of plant extract (0.3 μg/mL) and 5 μL of PPE (0.4725 U/mL). The resulting solutions were incubated for 15 min at room temperature avoiding light exposure. Afterwards, 15 μL of 2 mM N-succinyl-Ala-Ala-Ala-p-nitroanilide dissolved in Trizma buffer were added and the mixtures were incubated for 30 min at 37 °C. Elastatinal was used as a positive control. Experiments were performed in triplicate. Absorbances were measured at 405 nm using the Infinite 200 PRO series plate reader (Tecan GmbH, Crailsheim, Germany). The inhibition percentage of elastase was calculated using the following formula: [(A − B) − (C − D)]/(A − B) × 100, where A: Control (*w*/*o* sample), B: Blank (*w*/*o* sample, *w*/*o* tyrosinase), C: Sample, D: Blank sample (*w*/*o* elastase). 

### 2.7. High-Performance Thin-Layer Chromatography (HPTLC) Profiling

A Camag HPTLC instrumental setup was used for generating the metabolic profile of the various extracts. Extracts’ solutions were prepared (10 mg/mL) in ethyl acetate or methanol, depending on the solvent used for extraction. The samples of the extracts were applied onto 20 × 10 cm TLC plates (silica gel 60, F254, Merck, Darmstadt, Germany) using the Automatic TLC sampler (ATS4, CAMAG, Muttenz, Switzerland) under the control of the software platform vision CATS with the following standard settings: 15 tracks with 8 mm bands, 8 mm distance from the lower edge, 20 mm from the left and right edges, and 11.4 mm between the different tracks. The application volume of the samples varied depending on the different extraction methods. 

The plates were developed with an automatic development chamber (ADC2) using standard settings: 20 min chamber saturation with filter paper, 10 min of plate conditioning at 33% relative humidity (MgCl_2_), and 5 min of plate drying. Ethyl acetate, methanol, water, formic acid (50:10:7:1; *v*/*v*/*v*/*v*) was used as mobile phase. For visualization of the spots, the HPTLC plates were sprayed with sulfuric vanillin derivatization reagent (i.e., 5% *w*/*v* vanillin in MeOH/5% *v*/*v* H_2_SO_4_ in MeOH 1:1 *v*/*v*).

### 2.8. Ultra-Performance Liquid Chromatography High-Resolution Mass Spectrometry (UPLC-HRMS) Analysis

UPLC-HRMS analysis was performed on an AQUITY system (Waters) connected to a LTQ-Orbitrap XL hybrid mass spectrometer (Thermo Fisher Scientific, Bremen, Germany) equipped with an electrospray ionization (ESI) source and operated in negative mode. A UPLC separation gradient was developed to efficiently resolve all compounds for a qualitative analysis. The flow rate was set at 0.4 mL/min and the solvent system was (A) water 0.1% formic acid and (B) acetonitrile. The elution program was: 2% B for 2 min; 100% B in 18 min; hold for 2 min and then return to 2% B in 1 min. Column equilibration was performed for 4 min at the end of the run. The injection volume was set to 10 μL and samples were injected at 0.3 mg/mL in water-acetonitrile solution (1:1) on a Supelco Ascentis Express C18 (100 × 2.1 mm i.d, 2.7 μm particle size). The HRMS data were acquired in negative mode over 100–1000 *m*/*z* range. The MS profile was recorded in full scan mode (scan time = 1 micro scans and maximum inject time = 500 ms). The ESI conditions were as follow: capillary temperature 320 °C; capillary voltage −40 V; tube lens −120 V; ESI voltage 2.7 kV. Nitrogen was used as sheath gas (40 Au) and auxiliary gas (8 Au).

### 2.9. Cell Lines and Cell Culture Conditions

Human newborn foreskin (BJ) fibroblasts and B16-F10 mouse skin melanoma cells were purchased from the American Tissue Culture Collection (ATCC), while the human neonatal foreskin fibroblast strain AG01523 was obtained from the Coriell Institute for Medical Research. BJ cells were maintained in Dulbecco’s modified Eagle’s medium (DMEM; Thermo Fisher Scientific, Waltham, MA, USA), supplemented with 10% (*v*/*v*) fetal bovine serum (FBS), 2 mM glutamine and 1% non-essential amino acids, whereas AG01523 cells were cultured in DMEM containing 15% (*v*/*v*) FBS and 2 mM glutamine, and B16-F10 cells in DMEM containing 10% (*v*/*v*) FBS and 2 mM glutamine. All cells were maintained in a humidified environment of 5% CO_2_ and 37 °C. They were subcultured using a trypsin-citrate solution (0.25–0.3%, respectively), and they were tested periodically and found to be mycoplasma free.

### 2.10. Assessment of Cytotoxicity

The cells were plated in 96-well flat-bottomed microplates at a density of 7000 cells/well in serum-containing medium. After 18 h to ensure cell attachment, medium was changed to serum-free DMEM containing serial dilutions of the test extracts. Cultures incubated with the corresponding vehicle (DMSO) concentrations served as negative controls. Following a 72 h incubation, the medium was replaced with methylthiazolyldiphenyl-tetrazolium bromide (MTT; Merck KGaA, Darmstadt, Germany) dissolved at a final concentration of 1 mg/mL in serum-free, phenol-red-free DMEM (Biochrom) for a further 4 h incubation. Then, the MTT formazan was solubilized in 2-propanol and the optical density was measured using a FLUOstar Optima (BMG Labtech, Ortenberg, Germany) microplate reader at a wavelength of 550 nm (reference wavelength 650 nm). 

### 2.11. Intracellular ROS Assay

Cells were plated in 96-well clear-bottomed black microplates at a density of 7000 cells/well in serum-containing medium and were left to adhere for 18 h. Then, the test extracts were added diluted in serum-free, phenol-red-free DMEM at the highest non-cytotoxic concentration. After a 24 h incubation, 2′,7′-dichlorodihydrofluorescein diacetate (DCFH-DA; Merck KGaA, Darmstadt, Germany) was added at a final concentration of 10 μM for further 45 min. Then, the medium was replaced by phosphate buffered saline (PBS) or PBS supplemented with 100 μM H_2_O_2_, and, following a 15 min incubation, fluorescence emission was determined at 520 nm following excitation at 485 nm in a FLUOstar Optima microplate reader.

### 2.12. Induction of Premature Senescence by H_2_O_2_ and SA β-Gal Staining

Stress-induced premature senescence (SIPS) in relatively early passage proliferating cells was induced by exposing subconfluent cell cultures to serial short term oxidative stress, as previously described with minor modifications [30]. Briefly, BJ cells were exposed to the subcytotoxic concentration of 300 μM H_2_O_2_ (three exposures of 48 h each) in the presence or absence of the extracts. Control cells were kept under the same culture conditions without H_2_O_2_. After the last exposure, senescence-associated beta-galactosidase (SA-β-gal) staining was performed, as described earlier [31], and cells were viewed under phase contrast optics in a TS-100F NIKON inverted microscope in order to score positively stained cells.

### 2.13. Measurement of Cellular Tyrosinase Activity

In order to assess tyrosinase activity, B16-F10 cells were plated in 6-well plates and next day, they were incubated with the extracts (the concentration used for each extract is mentioned in Table 1) for 24 h. Afterwards, cells were lysed with 1% NP-40 lysis buffer (pH 6.8), as described before [32]. An amount of 20 μg of total protein was diluted in PBS and mixed with 5 mM L-DOPA (Merck KGaA, Darmstadt, Germany). Samples were set in triplicate in a 96-well plate and were incubated at 37 °C for 1 h. The absorbance was measured at 492 nm using the Infinite 200 PRO series plate reader (Tecan GmbH, Crailsheim, Germany).

### 2.14. Measurement of Proteasome Chymotrypsin-Like Activity

The chymotrypsin-like activity in young BJ skin fibroblasts was evaluated, as described previously [33]. Briefly, the hydrolysis of the fluorogenic peptide Suc-Leu-Leu-Val-Tyr-AMC (Enzo Life Sciences, Inc., NY, USA) was recorded in a Versa Fluor^TM^ Fluorometer System (Bio-Rad laboratories, Hercules, CA, USA) at excitation and emission wavelengths of 360 and 440 nm, respectively. The concentration used for each extract is mentioned in Table 1.

### 2.15. Measurement of Cathepsins B, L Activity

The activity of cathepsins B, L was measured in young BJ cells, as described previously [30], by recording the hydrolysis of the fluorogenic peptide z-FR-AMC (Enzo Life Sciences, Inc., Farmingdale, NY, USA) in the Versa Fluor^TM^ Fluorometer System (excitation, 360 nm; emission, 440 nm). The concentration used for each extract is mentioned in Table 1.

### 2.16. Sirtuin 1 Deacetylase Activity Assay

Sirtuin 1 (SIRT1) deacetylase activity was evaluated in the extracts at the concentration shown in Table 1, according to the manual instruction of SIRT1 Fluorometric Drug Discovery Kit (Enzo Life Sciences, Inc., Farmingdale, NY, USA). The measured fluorescence was directly proportional to deacetylation activity of the SIRT1 enzyme in the sample and was evaluated (excitation, 360 nm; emission, 460 nm) in the Infinite 200 PRO series plate reader (Tecan GmbH, Crailsheim, Germany).

### 2.17. Immunofluorescence Antigen Staining

For the evaluation of histone H2A.X phosphorylation (γH2A.X) on Ser139, cells grown on coverslips were incubated with the extracts at the concentration shown in Table 1 for 24 h. Afterwards, cells were fixed in 4% formaldehyde in PBS, permeabilized with 0.2% Triton X-100 and blocked with 3% BSA in PBS followed by labeling with primary antibody against γH2A.X (#2577, Cell Signaling Technology, Danvers, MA, USA) and secondary FITC-conjugated IgG antibody (JAC-711095152, Jackson ImmunoResearch Laboratories, Inc., Ely, UK). For visualizing nuclei and F-actin, cells were counterstained with 4′,6-diamidino-2-phenylindole dihydrochloride (DAPI) and Alexa Fluor Phalloidin (Thermo Fisher Scientific, Waltham, MA, USA), respectively. Samples were viewed at a NIKON C1 Confocal Laser Scanning Microscope.

### 2.18. MMP Activity Assay

Confluent human skin fibroblast cultures were washed twice with serum-free DMEM and incubated for 48 h in the presence or absence (control) of the highest non-cytotoxic concentration of each extract (Table 1) dissolved in serum-free DMEM. Conditioned medium was collected, centrifuged at 10,000× *g* for 10 min to remove cell debris, and stored at −80 °C until use. MMP activity of the supernatants was determined by incubation for 24 h at 37 °C with the fluorogenic substrate Dabcyl-Gaba-Pro-Gln-Gly-Leu-Glu-(EDANS)-AIa-Lys-NH2 (TNO211; Merck KGaA, Darmstadt, Germany) at a final concentration of 10 μM. Cleavage of the Gly-Leu bond by MMPs (as assessed by the reversal of EDANS fluorescence quenching by Dabcyl) was monitored at 480 nm after excitation at 340 nm, using a FLUOstar Optima microplate reader. TNO211 serves as a substrate for MMP-1, -2, -3, and -9 [34].

### 2.19. Assessment of Cell Protective Activity against UV-B Irradiation

Cells were plated in 96-well flat-bottomed microplates at a density of 7000 cells/well in serum-containing medium. After 18 h to ensure cell attachment, the medium was changed to serum-free, phenol red-free DMEM containing the highest non-cytotoxic concentrations of the extracts to be tested. Following further incubation for 24 h, cells were subjected to UV-B irradiation for 10 min (corresponding to 726 mJ/cm^2^) using a black box equipped with a closely spaced array of four UV-B lamps (Sankyo Denki, Hangzhou, Zhe Jiang, China) emitting between 280 nm and 360 nm (peak at 306 nm). Cytotoxicity was estimated after 72 h, using the MTT-method, as described above. Plates treated in identical manner, except for the UV-B irradiation, were used as controls.

### 2.20. Statistical Analysis

Experiments were performed at least in duplicates unless otherwise indicated in figure legends. For statistical analyses MS Excel was used. Statistical significance was evaluated using Student’s *t*-test (*t*-test). Data points correspond to the mean of the independent experiments and error bars denote standard deviation (SD.); significance at *p* < 0.05 or *p* < 0.01 is indicated in graphs by one or two asterisks, respectively.

## 3. Results

### 3.1. Selection and Extraction of Plant Species

The Greek flora is known to harvest a great number of plants with antioxidant and other therapeutic properties. In the herein study, 408 species and subspecies, almost 7% of the Greek flora, representing 66 families were collected (wild or cultivated) from diverse floristic regions of the country, covering large part of the geological variety and endemic flora of Greece. The plants were selected based on previous knowledge on ethnopharmacological information, chemodiversity and chemotaxonomy criteria and literature survey related to reported anti-aging activity of the genus/species.

The major goal was to include endemic plants of Greece, and finally 97 were included. It should be pointed out that the majority of plants belonged to the families Lamiaceae (91 species), Compositae (65 species), Leguminosae (58 species) and Umbelliferae (25 species). Thirty-six plant families were represented by one to two species, 15 families by three to five species, four families by six to seven species and four families by 11–12 species. In some cases, the same plant species were collected from different locations, while for some species different parts of the plant were separately extracted. In the Lamiaceae family, most of the species belonged to the genera: *Marrubium*, *Mentha*, *Nepeta*, *Origanum*, *Salvia*, *Satureja*, *Sideritis*, *Stachys*, *Teucrium* and *Thymus*. In the Compositae family, the genera with the highest representation were *Achillea*, *Centaurea* and *Inula*. *Astragalus* was the genus with the highest representation in the Leguminosae family and *Eryngium* in the Umbelliferae family. 

Ethyl acetate and methanol extracts of each plant material were prepared using ASE, as it provides a standardized and reproducible procedure, generating a focused extracts’ library. The extracts were subsequently screened for their antioxidant and anti-melanogenic potential, using filter concentrations, to step-wisely eliminate the inactive samples and discover the most promising candidates to afford bioactive secondary metabolites. The goal was to have a wealth of information from in vitro experiments (chemical and enzymatic) that could be combined and lead to the final selection of most promising plant extracts. 

### 3.2. Antioxidant and Anti-Melanogenic Activities of Plant Extracts

Initially, their free radical scavenging potential was investigated by employing the DPPH assay. Of the plant extracts, 167 showed a high scavenging activity (>50%), with 99 of them showing an inhibition >80% (Supplementary Table S2). Almost 85% were methanolic extracts and mainly belonged to the Lamiaceae family. 

The 167 plant extracts with DPPH-scavenging activity >50% were tested for their cytotoxic activity on human skin fibroblasts. The cells were incubated with the extracts for a period of 72 h, and then cytotoxicity was assessed using the MTT assay. Of the extracts, 74 were not cytotoxic at the highest concentration tested, i.e., 100 μg/mL, while for the rest the highest non-cytotoxic concentration was determined to be employed in all further cell-based assays (Supplementary Table S2). Furthermore, all 167 plant extracts at their highest non-cytotoxic concentration were also tested for their capacity to scavenge intracellular ROS both in cultures challenged with H_2_O_2_ and unchallenged ones (Supplementary Table S2), since we sought to verify that the extracts’ antioxidant activity was evident in the cellular context, i.e., the extracts can be internalized in the cytoplasm and act as antioxidants intracellularly. In general, there was no correlation between the DPPH-scavenging activity and the intracellular-ROS attenuation capacity of the extracts, both in H_2_O_2_-challenged cultures and in unchallenged ones. This may reflect the diversity of the studied extracts leading to a variety of cell internalization degrees, as we have previously observed a relatively good correlation between the DPPH and ROS assays in a rather uniform set of extracts [35].

In parallel, all extracts were tested for their anti-melanogenic properties through the evaluation of their inhibitory capacity against diphenolase activity of mushroom tyrosinase (Supplementary Table S2). The extracts showed a variety of inhibition activity with 20% of them, showing an inhibitory activity of >40%. Of the extracts, 61% were produced with methanolic extraction. *Glycyrrhiza glabra*, *Paeonia mascula* subsp. *hellenica*, *Sedum sediforme* and *Umbilicus horizontalis* showed an increased inhibitory activity. Generally, the families showing the highest tyrosinase inhibitory activity were the Lamiaceae and the Leguminosae.

In order to proceed with further biological assays, extracts exhibiting both antioxidant and anti-melanogenic properties (DPPH scavenging activity >80% and anti-tyrosinase activity >40%) were considered the most promising and thus were selected for further evaluation. The extracts with the most potent antioxidant and anti-melanogenic properties of Greek flora as emerged through these extensive screening assays were produced from methanolic extraction of the following plants: *Satureja hortenis* (EXT1), *Origanum majorana* (EXT2), *Origanum vulgare* ssp. *hirtum* (EXT3), *Hyssopus officinalis* (EXT4), *Salvia fruticosa* (EXT5), *Salvia pomifera* (EXT6), *Salvia officinalis* (EXT7), *Salvia sclarea* (EXT8), *Sideritis scardica* (EXT9), *Melissa officinalis* (EXT10), *Thymus vulgaris* (EXT11)*, Hypericum perforatum* (EXT12), *Glycyrrhiza glabra* (EXT13), *Geranium macrorrhizum* (EXT14), *Umbilicus horizontalis* (EXT15), *Sedum sediforme* (EXT16), *Paeonia mascula* (EXT17) and *Rosa damascena* (EXT18) (Table 1).

These prominent extracts were further tested for their hyaluronidase and elastase inhibition activities and the results are presented in Table 2.

As shown in Table 2, two plant extracts, *S. sediforme* and *U. horizontalis,* exhibited a high inhibitory activity in both assays, while *H. perforatum*, *S. sclarea* and *S. scardica* showed low activity in both assays. *S. pomifera*, *S. fruticosa*, *T. vulgaris*, *G. macrorrhizum*, *G. glabra* and *H. officinalis* had a medium inhibitory activity in both assays. *M. officinalis*, *O. majorana*, *O. vulgare* subsp. *hirtum*, *P. mascula*, *R. damascena*, *S. officinalis* and *S. hortenis* showed medium to good inhibitory activity at only one assay.

### 3.3. Phytochemical Profile of the Extracts

**Figure 1:**
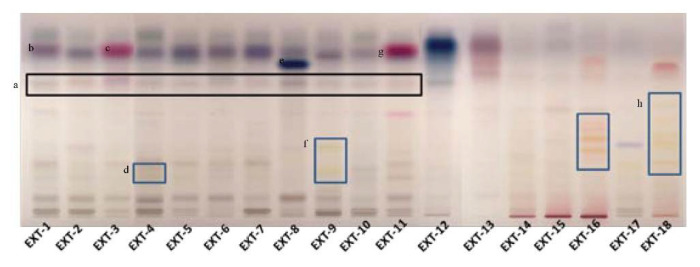
Phytochemical investigation of selected plant extracts with HPTLC (**a**: the black box marks the rosmarinic acid, **b**: α-amyrin, **c**: thymol/carvacrol, **d**: flavonoid glucosides, **e**: salvianolic acid, **f**: flavonoid glucosides, **g**: thymol/carvacrol, **h**: flavonoid glucosides).

The most promising extracts were further analyzed for their phytochemical profile with HPTLC as shown in Figure 1.

The phytochemical content of the examined plants’ extracts was found to be rich, containing a variety of compounds, from phenolic substances to terpenoids. The presence of rosmarinic acid was evident in the plant species, belonging to the Lamiaceae family (Figure 1). In *S. hortensis* as well as *M. officinalis*, the occurrence of phenolic acid derivatives was characteristic in low Rf. In *S. hortensis*, α-amyrin was also identified by the characteristic color and Rf. In places 2 and 3, *Origanum* extracts were developed. The presence of phenolic acids is characteristic with hydroxycinnamic acid and hydroxybenzoyl acid derivatives. In the case of *O. vulgare*, compounds (thymol/carvacrol) that occur in the essential oil are identified, indicating its aromatic character. Rosmarinic acid was also identified in the extract of *H. officinalis* along with flavonoid glucosides. It is characteristic that plant species belonging to the genus *Salvia* shared a common chemical fingerprint, *S. officinalis*, *S. pomifera*, *S. sclarea* and *S. fruticosa*, with phenolic acids, flavonoids and terpenoids. Quantitative differences between the species could be distinguished. A band with characteristic color seems to be in a higher amount in *S. sclarea* compared to the other species, possibly corresponding to a salvianolic acid component. *S. scardica* was also rich in flavonoid glucosides. The appearance of thymol/carvacrol was characteristic in the plate, while phenolic acid derivatives were also identified in lower Rf in the case of *T. vulgaris*. The HPTLC analysis in the case of *H. perforatum* showed the presence of naphthodianthrones (pseudohypericin and hypericin). Glycyrrhetinic acid prevailed in the extract of *G. glabra*. Phenolic acids’ derivatives were the major constituents in *G. macrorrhizum* and *U. horizontalis*. *S. sediforme* was rich in flavonoids, possibly derivatives of eriodictyol and myricetin glucosides. The analysis of *P. mascula* showed the presence of tannins as well as flavonoids. The extract of *R. damascena* was rich in flavonoid glucosides.

### 3.4. Intracellular Antioxidant Activity of the Extracts

**Figure 2:**
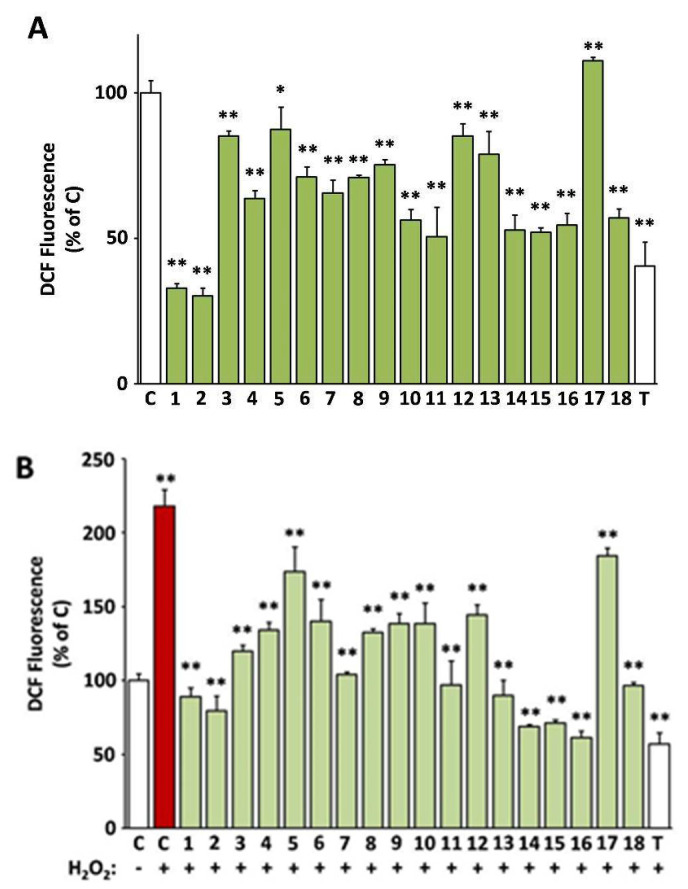
Intracellular antioxidant activity of the extracts. 2′,7′-dichlorofluorescein (DCF)-fluorescence of human skin fibroblast cultures (AG01523) treated with the indicated extracts for 24 h was used for the assessment of basal (**A**) or H_2_O_2_-stimulated reactive oxygen species (ROS) levels (**B**). Vehicle (dimethyl sulfoxide, DMSO) and Trolox were used as negative (C) and positive (T) controls, respectively. Bars ± SD. * *p* < 0.05; ** *p* < 0.01. Control (C) is set to 100%.

In Figure 2, the capacity of the 18 selected extracts to scavenge intracellular ROS both in cultures challenged with H_2_O_2_ and unchallenged ones is presented (values are also indicated in Supplementary Table S2). All extracts except from EXT17 were capable of reducing the levels of the intracellular ROS, as assessed using the DCFH-DA method. The most active extracts EXT2 and EXT1 (*O. majorana* and *S. hortensis*, respectively) exhibited more intense ROS level attenuation than the compound used as positive control, i.e., Trolox at 20 μg/mL (79.9 μM). The antioxidant activity of the extracts was also tested in the presence of an external oxidative stimulus, i.e., hydrogen peroxide (H_2_O_2_) at a concentration of 100 μM, which was not cytotoxic. As shown in Figure 2B, H_2_O_2_ induced a 2.2-fold induction of ROS levels in human skin fibroblasts, which was prevented by pre-incubation with all test extracts, even EXT17 (*P. mascula*, which was inactive regarding basal ROS levels). The most active extracts regarding the suppression of the exogenous ROS induction were EXT16, EXT14, and EXT15 (*S. sediforme*, *G. macrorrhizum*, and *U. horizontalis*, respectively), the first ones being equipotent to Trolox (20 μg/mL).

### 3.5. Cell-Based Evaluation of the Anti-Melanogenic Activity of the Extracts

**Figure 3:**
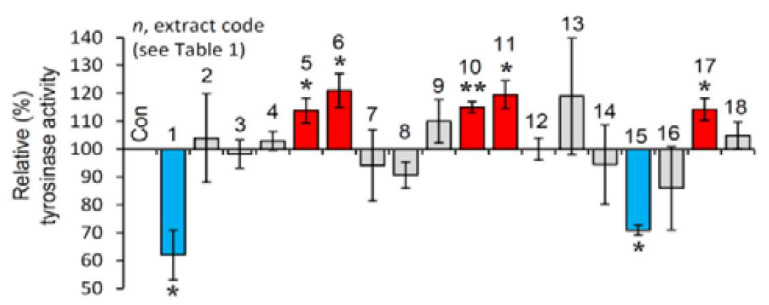
Cell-based anti-melanogenic activity of the extracts. Relative (%) tyrosinase activity in B16-F10 melanocytes treated with the extracts for 24 h. Dimethyl sulfoxide (DMSO)-treated cultures served as controls (con). Bars ± SD (*n* ≥ 2). * *p* < 0.05; ** *p* < 0.01. Control values were set to 100%.

The selected extracts were then examined for their effect on tyrosinase activity in B16-F10 melanocytes. As observed in Figure 3, and in line with the cell-free findings, the extracts EXT1 and EXT15 were found to exhibit significant anti-melanogenic activity.

### 3.6. Effect of the Extracts on Sirtuin 1 Enzymatic Activity

**Figure 4:**
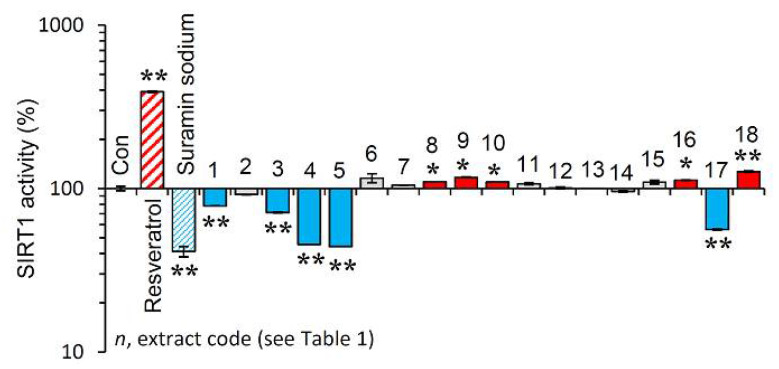
Effect of the plant extracts on Sirtuin 1 enzymatic activity. Relative (%) Sirtuin 1 activity of the extracts. Resveratrol and suramin sodium, a Sirtuin 1 activator and inhibitor, respectively, were used as positive controls. Dimethyl sulfoxide (DMSO) served as control (con). Bars ± SD. (*n* ≥ 2). * *p* < 0.05; ** *p* < 0.01. Control values were set to 100%.

Afterwards, we examined the effect of the extracts on the activity of SIRT1. Reportedly, sirtuins are key modules affecting the process of aging, as their overexpression seems to mimic caloric restriction leading thus to lifespan extension in most invertebrate model organisms [19]. As shown in Figure 4, the extracts EXT8, EXT9, EXT10, EXT16 and EXT18 were found to be activators of SIRT1 enzymatic activity.

### 3.7. Effect of the Extracts on the Main Proteostatic Pathways

**Figure 5:**
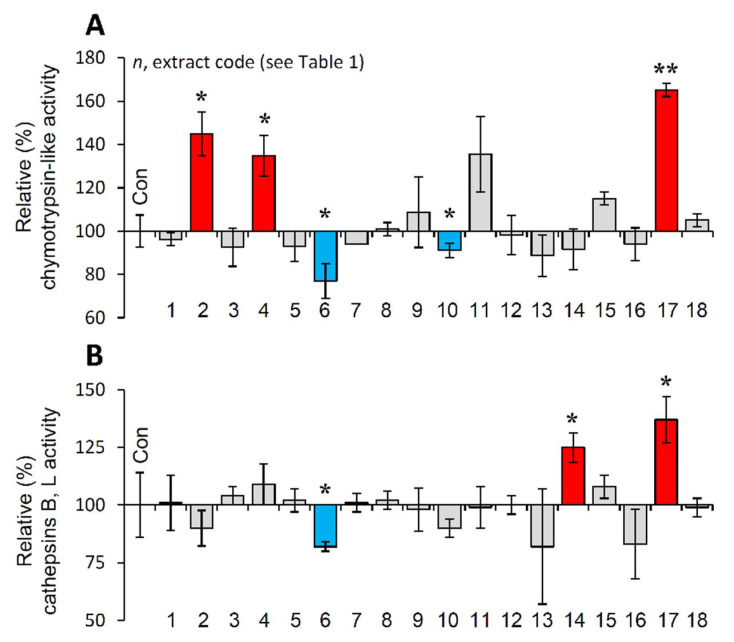
Effect of the extracts on the main proteostatic pathways. (**A**) Relative (%) chymotrypsin-like (CT-L) activity after incubation of young BJ cells with the extracts for 24 h. (**B**) Relative (%) cathepsins B, L activity in BJ cells treated with the extracts for 24 h. Dimethyl sulfoxide (DMSO)-treated cultures served as controls (con). Bars ± SD. (*n* ≥ 2). * *p* < 0.05; ** *p* < 0.01. Control values were set to 100%.

It is evident that PN functionality declines during cellular senescence or in vivo aging and the activation of proteostatic modules is proposed to exert anti-aging effects [10,20]. Thus, the most promising extracts were further examined for their capacity to activate cellular protective mechanisms. We observed that the extracts EXT2, EXT4 and EXT17 increased the main proteasome peptidase activity, i.e., the chymotrypsin-like activity (CT-L/β5) in human skin fibroblasts (Figure 5A). Furthermore, cell exposure to the extracts EXT14 and EXT17 led to significant induction of cathepsins B, L enzymatic activity (Figure 5B).

### 3.8. Protection of Cells against Oxidative Stress-Mediated Premature Senescence by the Extracts

**Figure 6:**
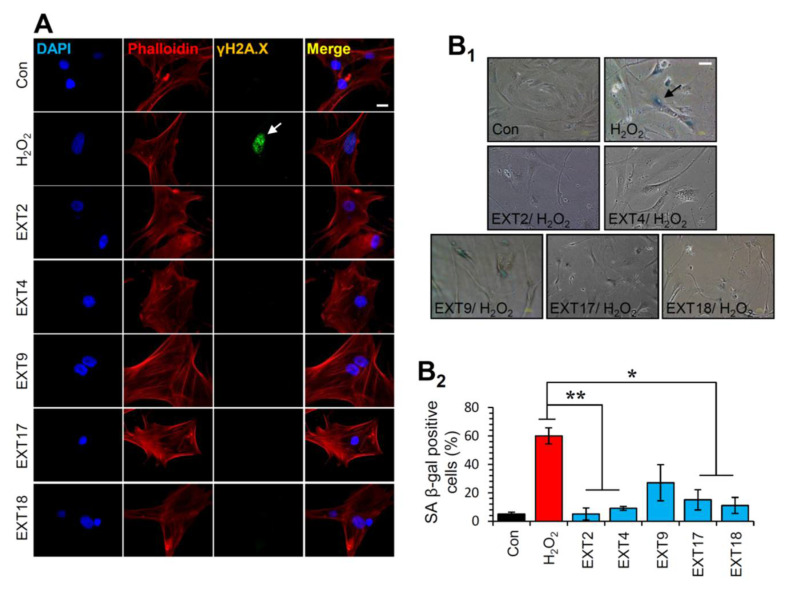
Protection of cells against oxidative stress-mediated premature senescence by the extracts. (**A**) Immunofluorescence images following detection of the phosphorylation form of H2A.X (γH2A.X) on Ser139 in BJ cells incubated with the indicated extracts for 24 h; cells nuclei were counterstained with DAPI and Phalloidin. (**B1**) Representative light field images following SA β-gal staining of control BJ cells or cells treated (three exposures of 48 h each) with 300 μM H_2_O_2_ in the presence or absence of the extracts. (**B2**) Relative (%) number of SA β-gal positive cells (mean of 10 optical fields) following treatment of cells exactly as in (B1). Bars ± SD. * *p* < 0.05; ** *p* < 0.01. Bars, in (**A**) 10 μM and in (**B1**) 100 μM.

Thereafter, the most promising extracts, i.e., EXT2, EXT4, EXT9, EXT17 and EXT18 were further investigated for any genotoxic impact on BJ cells. To this end, we assessed the phosphorylation of H2A.X on Ser139, which is a marker of DNA damage [36]. As shown in Figure 6A, immunofluorescence data for γH2A.X revealed that treatment of cells with the extracts for 24 h did not cause any DNA damage. The formation of phosphorylated H2A.X foci in the nuclei of the cells was detected in the cells treated with 300 μΜ H_2_O_2_ (positive control) for 24 h (Figure 6A).

Given the aforementioned findings, we then sought to examine whether the extracts could protect cells from SIPS. Thus, SIPS was induced by exposing BJ cells to H_2_O_2_ in the presence or absence of the extracts and the percentage of SA β-gal positive cells was counted. Interestingly, we found that the extracts EXT2, EXT4, EXT17 and EXT18 conferred significant protection against H_2_O_2_-mediated SIPS. The extract EXT9 tended to protect cells from SIPS; however, the decline of the SA-β-gal positive cells in the presence of the extract (compared to control cells) was not statistically significant (Figure 6B).

### 3.9. Suppression of the Secreted Matrix Metalloproteinases (MMP)-Activity by the Extracts

**Figure 7:**
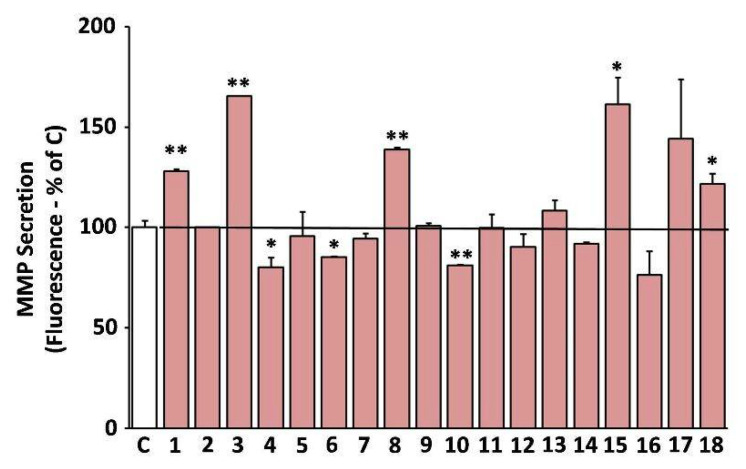
Secreted matrix metalloproteinases (MMP) activity after treatment with the extracts. Media conditioned by human skin fibroblasts (AG01523) treated with the indicated extracts for 48 h were assessed for their MMP activity. Conditioned medium from dimethyl sulfoxide (DMSO)-treated culture served as negative control (C). Bars ± SD. * *p* < 0.05; ** *p* < 0.01. Control set to 100%.

A shift of the cell secretome towards catabolism of the ECM is a well-known hallmark of cellular senescence [37]. Furthermore, induction of the ECM degrading MMPs is a key feature of skin photoaging [16]. Hence, we studied the effects of the extracts on the MMP-activity secreted by human skin fibroblast cultures in their supernatant (conditioned medium). Some of the extracts were observed to stimulate the secreted MMP activity, such as EXT3, EXT18, EXT8, EXT1, and EXT15 (*O. vulgare*, *R. damascena*, *S. sclarea*, *S. hortensis*, and *U. horizontalis*, respectively; Figure 7). On the other hand, EXT4, EXT10, and EXT6 (*H. officinalis*, *M. officinalis*, and *S. pomifera*, respectively) statistically significantly suppressed the MMP-activity in human skin fibroblast conditioned medium.

### 3.10. Protective Activity of the Extracts against UV-B Cytotoxicity

**Figure 8:**
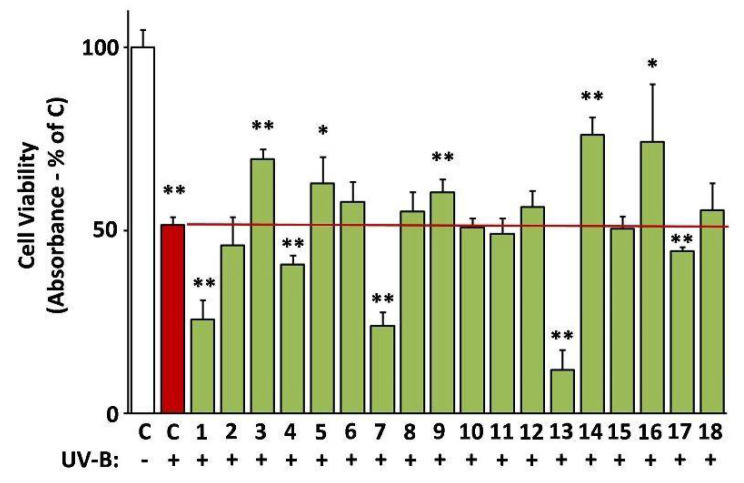
Photoprotective activity of the extracts. UV-B-induced cytotoxicity was assessed on human skin fibroblasts (AG01523) pre-treated with the indicated extracts for 24 h. Dimethyl sulfoxide (DMSO)-treated cultures served as controls (C). UV-B-untreated control set to 100%. Bars ± SD. * *p* < 0.05; ** *p* < 0.01.

The ability of the extracts to protect against UV-B radiation was assessed based on their capacity to reverse UV-B-induced death of human skin fibroblasts [24]. Indeed, EXT14, EXT3, EXT5, EXT9, and EXT16 (*G. macrorrhizum*, *O. vulgare*, *S. fruticosa*, *S. Scardica*, and *S. sediforme*, respectively), provoked a statistically significant inhibition of UV-B cytotoxicity (Figure 8).

**Figure 9:**
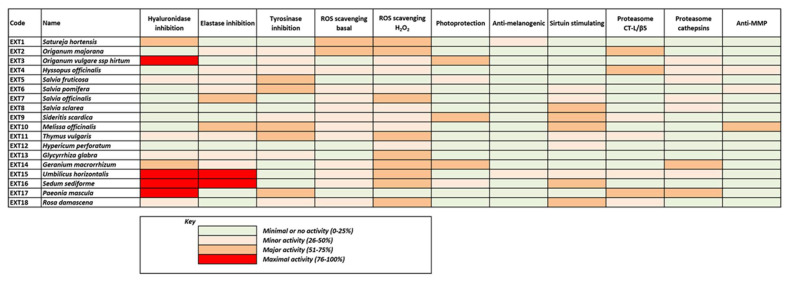
Combined colored visualization of the extracts’ bioactivities based on the results presented in Figure 2, Figure 3, Figure 4, Figure 5, Figure 6, Figure 7 and Figure 8 and Table 1 and Table 2.

Most of the above results are illustrated collectively in Figure 9, where the heterogeneity of the extracts’ bioactivities is obvious, also reflecting the diversity of their phytochemical content. Although few of the extracts exhibit maximal bioactivities in certain assays, all of them seem to combine considerable activities in many assays. This fact renders all these extracts significant candidates for use in cosmetic products against skin aging.

### 3.11. LC-MS Analyses of Selected Extracts

**Figure 10:**
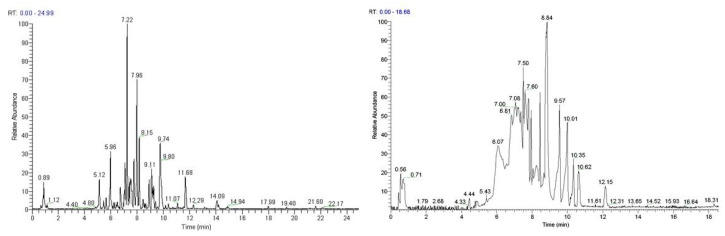
Base peak chromatogram of the *S. scardica* (left) and *R. damascena* (right) methanolic extracts, negative ionization mode.

Based on their total profiles, *S. scardica* and *R. damascena* methanolic extracts were chosen to be analyzed by UPLC-HRMS and their metabolites were qualitatively characterized. Table 3 and Table 4 include the compounds identified in the extracts and Figure 10 the base peak chromatograms (BPCs) of the analyzed extracts. Metabolites were assigned by interpreting the mass spectra determined through their MS, considering all the data provided by the literature. Twenty-four compounds were identified in the *S. scardica* extract. As seen, the extract is rich in phenolic compounds, mainly flavonoids and phenylethanoid glycosides, as was also observed with HPTLC. Among them, verbascoside, isoscutellarein 7-*O*-[6‴-*O*-acetyl]-allosyl(1→2)-glucoside, 4′-*O*-methylhypolaetin 7-*O*-[6‴-*O*-acetyl]-allosyl(1→2)-glucoside and apigenin-(coumaroylglucoside) seem to be the main compounds identified in the extract. Eighteen compounds were detected in the methanolic extract of *R. damascena*. In this case also, flavonoids prevailed, in accordance with HPTLC analysis. Rosmarinic acid and kaempferol 3-*O*-(6″-*O*-E-π-coumaroyl)-β-D-glucopyranoside were identified as the main peaks.

The above results are in accordance with previous works identifying verbascoside, isoscutellarein-7-*O*-[allosyl (1→2) glucoside] and apigenin-(coumaroylglucosides) in various species of the *Sideritis* genus [38,39,40]. More importantly, our results regarding the photoprotective and anti-aging effects of the *S. scardica* extract (EXT 9, see Figure 4, Figure 8, and Figure 9) are also in line with reports attributing such properties in verbascoside (also known as acteoside) [41,42,43,44], which from Figure 10 seems to be the most abundant constituent of EXT 9. In line with the anti-aging features of the *R. damascena* extract (EXT 18, see Figure 4, Figure 6, and Figure 9) presented in the current study, a methanolic [45] and an aqueous [46] extract of this plant were found to extend the lifespan of the fruit fly. Furthermore, our findings are in accordance with observations regarding the protective effects of rosmarinic acid—the second most abundant constituent of EXT 18—against cellular senescence [47,48,49,50]. Kaempferol and its derivatives have also been recently reported to act as senomorphic agents [51].

## 4. Conclusions

The present high-throughput screening of more than 440 plant species and subspecies representing almost 7% of the Greek flora was based on the antioxidant and anti-melanogenic properties of the plant extracts. The most promising 4% of the extracts were analyzed further, and they were subjected to a battery of in vitro and cell-based assays covering most of the parameters contributing to skin aging. *S. scardica* and *R. damascena* methanolic extracts were analyzed in detail by UPLC-HRMS. Most of the selected extracts may be of great value for the cosmetic industry, since they combine antioxidant, photoprotective, anti-melanogenic and anti-aging properties.

## Figures and Tables

**Table 1:** Extracts selected for further biological assays.

Plant Species	Code	Highest Non-Cytotoxic Concentration (μg/mL)
*Satureja hortensis*	EXT1	100
*Origanum majorana*	EXT2	20
*Origanum vulgare* subsp. *hirtum*	EXT3	100
*Hyssopus officinalis*	EXT4	20
*Salvia fruticosa*	EXT5	4
*Salvia pomifera*	EXT6	20
*Salvia officinalis*	EXT7	20
*Salvia sclarea*	EXT8	20
*Sideritis scardica*	EXT9	20
*Melissa officinalis*	EXT10	100
*Thymus vulgaris*	EXT11	20
*Hypericum perforatum*	EXT12	4
*Glycyrrhiza glabra*	EXT13	20
*Geranium macrorrhizum*	EXT14	100
*Umbilicus horizontalis*	EXT15	100
*Sedum sediforme*	EXT16	100
*Paeonia mascula*	EXT17	0.16
*Rosa damascena*	EXT18	20

**Table 2:** Hyaluronidase and Elastase inhibition activity of selected extracts.

Extract	Hyaluronidase Inhibition (% at 300 μg/mL)	Elastase Inhibition (% at 300 μg/mL)
*Geranium macrorrhizum*	51.09	46.64
*Glycyrrhiza glabra*	35.81	48.75
*Hypericum perforatum*	8.86	−4.24
*Hyssopus officinalis*	24.73	28.20
*Melissa officinalis*	−0.98	58.02
*Origanum majorana*	11.27	41.14
*Origanum vulgare* subsp. *hirtum*	81.04	1.01
*Paeonia mascula*	99.67	5.80
*Rosa damascena*	27.52	17.28
*Salvia officinalis*	8.57	55.31
*Salvia pomifera*	23.07	40.40
*Salvia sclarea*	−6.01	−14.56
*Salvia fruticosa*	28.93	42.71
*Satureja hortensis*	52.92	−26.38
*Sedum sediforme*	98.49	89.07
*Sideritis scardica*	13.95	−19.95
*Thymus vulgaris*	31.07	31.18
*Umbilicus horizontalis*	89.04	87.84

**Table 3:** Secondary metabolites identified in the *S. scardica* methanolic extract.

No.	Compounds	Rt (min)	Theoretical	Experimental	Δm (ppm)	RDBeq	Molecular Formula
			[M-H]^−^ *m*/*z*				
1	Feruloylquinic acid derivative	0.84	367.1035	367.1021	0.310	8.5	C_17_H_20_O_9_
2	Melittoside derivative	1.12	569.1719	569.1709	0.069	15.5	C_29_H_30_O_12_
3	5-Caffeoylquinic acid	5.96	353.0870	353.0855	0.259	8.5	C_16_H_18_O_9_
4	Apigenin 7-*O*-allosyl(1→2)-glucoside	6.28	593.1492	593.1472	0.189	13.5	C_27_H_30_O_15_
5	Echinacoside	7.01	785.2484	785.2475	0.465	13.5	C_35_H_46_O_20_
6	Lavandulifolioside	7.08	755.2380	755.2360	1.189	13.5	C_34_H_44_O_19_
7	Verbascoside	7.22	623.1981	623.1981	0.598	12.5	C_29_H_36_O_15_
8	Hypolaetin 7-*O*-allosyl(1→2)-glucoside	7.48	625.2020	625.2015	1.100	13.5	C_31_H_34_O_18_
9	Samioside	7.08	755.2404	755.2402	0.254	13.5	C_34_H_44_O_19_
10	Isoscutellarein 7-*O*-allosyl(1→2)-glucoside	7.38	609.1461	609.1459	0.456	13.5	C_27_H_30_O_16_
11	Allysonoside	7.59	769.2561	769.2555	0.278	13.5	C_35_H_46_O_19_
12	Hypolaetin 7-*O*-[6‴-*O*-acetyl]-allosyl(1→2)-glucoside	7.52	667.1516	667.1511	0.512	14.5	C_29_H_32_O_18_
13	Leucosceptoside A	7.74	637.2138	637.2125	0.357	12.5	C_30_H_38_O_15_
14	3′-*O*-Methylhypolaetin 7-*O*-allosyl(1→2)-glucoside	7.56	639.1567	639.1561	0.632	13.5	C_28_H_32_O_17_
15	Apigenin 7-*O*-glucoside	7.79	431.0984	431.0979	0.558	12.5	C_21_H_20_O_10_
16	Isoscutellarein 7-*O*-[6‴-*O*-acetyl]-allosyl(1→2)-glucoside	7.96	653.1618	653.1612	0.234	13.5	C_29_H_32_O_17_
17	Apigenin 7-*O*-[6‴-*O*-acetyl]-allosyl(1→2)-glucoside	7.88	653.1618	635.1610	0.589	14.5	C_29_H_32_O_16_
18	Isoscutellarein 7-*O*-allosyl-(1→2)-[6″-*O*-acetyl]-glucoside	7.96	651.1567	651.1565	0.676	14.5	C_29_H_32_O_17_
19	4′-*O*-Methylhypolaetin 7-*O*-[6‴-*O*-acetyl] -allosyl(1→2)-glucoside	8.15	681.1672	681.1669	1.347	14.5	C_30_H_34_O_18_
20	3′-*O*-Methylhypolaetin 7-*O*-[6‴-*O*-acetyl]-allosyl-(1→2)-[6″-*O*-acetyl]-glucoside or 4′-*O*-Methylhypolaetin 7-*O*-[6‴-*O*-acetyl] -allosyl-(1→2) [6″-*O*-acetyl]-glucoside	9.27	723.1767	723.1765	0.457	15.5	C_32_H_36_O_19_
21	4′-*O*-Methylisoscutellarein 7-*O*-[6‴-*O*-acetyl]-allosyl(1→2)-glucoside	9.15	665.1723	665.1720	0.968	14.5	C_30_H_34_O_17_
22	Isoscutellarein 7-*O*-[6‴-*O*-acetyl]-allosyl(1→2)-[6″-*O*-acetyl]-glucoside	9.11	693.1672	693.1668	0.436	15.5	C_31_H_34_O_18_
23	Apigenin	9.78	269.0455	269.0450	0.969	11.5	C_15_H_10_O_5_
24	Apigenin 7-(6″-p-coumaroylglucoside) or Apigenin 7-(4″-p-coumaroylglucoside)	9.74	577.1351	577.1348	0.336	18.5	C_30_H_26_O_12_

**Table 4:** Secondary metabolites identified in *R. damascena* methanolic extract.

No.	Compounds	Rt (min)	Theoretical	Experimental	Δm (ppm)	RDBeq	Molecular Formula
			[M-H]^−^ *m*/*z*				
1	Quinic acid	0.65	165.0405	165.0408	1.972	1.5	C_7_H_12_O_6_
2	Gallic acid	2.69	169.0142	169.0147	0.753	5.5	C_7_H_6_O_5_
3	Protocatechuic acid	4.70	153.0193	153.0196	2.000	5.5	C_7_H_6_O_5_
4	Quercetin 3-*O*-glucoside	6.07	463.0882	463.0885	0.718	12.5	C_21_H_20_O_12_
5	Rutin	6.57	609.1461	609.1462	0.217	13.5	C_27_H_30_O_16_
6	Quercetin 3-*O*-pentoside	7.04	433.0776	433.0778	0.357	12.5	C_20_H_18_O_11_
7	Hyperoside	7.08	463.0882	463.0883	0.322	12.5	C_21_H_20_O_12_
8	Quercetin-glucuronide	7.08	477.0675	477.0674	−0.042	13.5	C_21_H_18_O_13_
9	Quercetin 3-*O*-rhamnoside	7.22	447.0933	447.0934	0.427	12.5	C_21_H_20_O_11_
10	Kaempferol 3-*O*-glucoside	7.22	447.0933	447.0934	0.1537	12.5	C_21_H_20_O_11_
11	Kaempferol-3-*O*-rutinoside	7.26	593.1512	593.1513	0.190	13.5	C_27_H_30_O_15_
12	Rosmarinic acid	7.50	359.0772	359.0773	0.253	11.5	C_18_H_16_O_8_
13	Caffeic acid	7.60	179.0350	179.0354	2.251	6.5	C_9_H_8_O_4_
14	Kaempferol pentoside	7.70	417.0827	417.0828	0.178	12.5	C_20_H_18_O_10_
15	Kaempferol-3-*O*-rhamnoside	8.05	431.0984	431.0984	0.1142	12.5	C_21_H_20_O_10_
16	Kaempferol 3-*O*-(6″-*O*-E-π-coumaroyl)-β-D-glucopyranoside	8.84	593.1301	593.1302	0.209	18.5	C_30_H_26_O_13_
17	Quercetin	8.95	301.0354	301.0354	0.183	11.5	C_15_H_10_O_7_
18	Kaempferol	9.57	285.0405	285.0404	−0.197	11.5	C_15_H_10_O_6_

## Data Availability

Data is contained within the article or supplementary material.

## Supplementary Materials

The following are available online at https://www.mdpi.com/article/10.3390/antiox10081206/s1, Table S1: Samples of plants studied. The botanical name, family, plant parts used and place of collection are presented. “*” shows the endemic plant species, Table S2: Initial evaluation of plant extracts.
