# Supplementary material for: Phytochemical Study and In Vitro Screening Focusing on the Anti-Aging Features of Various Plants of the Greek Flora
Source: Antioxidants (Basel). 2021 Jul 28;10(8):1206. doi: 10.3390/antiox10081206 (PMC8389045; doi:10.3390/antiox10081206)
Supplement: Supplementary file 1 [file antioxidants-10-01206-s001.zip › antioxidants-1296160-supplementary.pdf]

Supplementary Information:

Phytochemical Study and *In Vitro* Screening Focusing on the Anti-Aging Features of Various Plants of the Greek Flora

Aimilia D. Sklirou [1,†], Maria T. Angelopoulou [2,†], Aikaterini Argyropoulou [3,†], Eliza Chaita [3], Vasiliki Ioanna Boka [3], Christina Cheimonidi [1], Katerina Niforou [1], Eleni Mavrogonatou [2], Harris Pratsinis [2], Eleftherios Kalpoutzakis [3], Nektarios Aligiannis [3], Dimitris Kletsas [2], Ioannis P. Trougakos [1] and Alexios Leandros Skaltsounis [3,4] *

[1] Department of Cell Biology and Biophysics, Faculty of Biology, National and Kapodistrian University of Athens, Athens, 15784, Greece;
asklirou@biol.uoa.gr (A.D.S.); chrischeim@biol.uoa.gr (C.C.); kniforou@biol.uoa.gr (K.N.); itrougakos@biol.uoa.gr (I.P.T.)
[2] Laboratory of Cell Proliferation and Ageing, Institute of Biosciences and Applications, NCSR ''Demokritos'', Athens, 15310, Greece;
mangelopoulou@bio.demokritos.gr (M.T.A.); elmavro@bio.demokritos.gr (E.M.); hprats@bio.demokritos.gr (H.P.); dkletsas@bio.demokritos.gr (D.K.)
[3] Division of Pharmacognosy and Natural Products Chemistry, Department of Pharmacy, National and Kapodistrian University of Athens, Panepistimiopolis Zografou, Athens, 15771, Greece;
katarg@pharm.uoa.gr (A.A.); elchaita@pharm.uoa.gr (E.C.); vana_b@otenet.gr (V.I.B.); elkalp@pharm.uoa.gr (E.K.); aligiannis@pharm.uoa.gr (N.A.); skaltsounis@pharm.uoa.gr (A.L.S.)
[4] Institute of Organic and Analytical Chemistry (ICOA), UMR 7311, Université d'Orléans, rue de Chartres, Orléans, 45067, France
* Correspondence: itrougakos@biol.uoa.gr; dkletsas@bio.demokritos.gr; skaltsounis@pharm.uoa.gr
† All three authors contributed equally to this manuscript

Supplementary Table 1. Samples of plants studied. The botanical name, family, plant parts used and place of collection are presented. "*" shows the endemic plant species.

Plant Species	Family	Plant part	Place of collection
Acantholimon androsaceum *	Plumbaginaceae	aerial parts	Crete island
Acanthus spinosus	Acanthaceae	aerial parts	Pateras mountain, Attiki
Achillea abrotanoides	Compositae	aerial parts	Tymfi mountain
Achillea absinthoides *	Compositae	flowering aerial parts and some perennial parts	Tzoumerka mountain
Achillea cretica	Compositae	aerial parts	Idi mountain, Crete
Achillea fraasii	Compositae	aerial parts	Tymfi mountain
Achillea maritima	Compositae	aerial parts	Attiki
Achillea millefolium	Compositae	aerial parts	Attiki
Achillea pindicola subsp. *intergifolia* *	Compositae	annual and perennial flowering aerial parts and some roots	Tzoumerka mountain
Achillea taygetea *	Compositae	aerial parts	Taygetos mountain
Achillea umbellata *	Compositae	aerial parts	Kyllini mountain
Acinos alpinus subsp. *alpinus*	Lamiaceae	flowering aerial parts and the roots	Tzoumerka mountain
Acinos alpinus subsp. *meridionalis*	Lamiaceae	flowering aerial parts	Taygetos mountain
Acinos suaveolens	Lamiaceae	flowering aerial parts	Oiti mountain
Agrimonia eupatoria	Rosaceae	aerial parts	Gkiona mountain
Ajuga orientalis	Lamiaceae	flowering aerial parts	Parnassos mountain
Alkana pindicola subsp. *pindicola*	Boraginaceae	aerial parts	Giona mountain
Allium ampeloprasum	Liliaceae	basal leaves and bulb	Zaros mountain-Crete
Alyssum saxatile	Cruciferae	aerial parts	Kastraki hill, Astros, Arkadia
Alyssum smolikanum	Cruciferae	flowering aerial parts	Smolikas mountain
Alyssum taygeteum *	Cruciferae	whole plant with flowers and fruits	Giona mountain
Amelanchier ovalis	Rosaceae	aerial parts	Parnassos mountain
Amelanchier parviflora subsp. *chelmea* *	Rosaceae	aerial parts	Zarax
Anagyris foetida	Leguminosae	fruits and seeds	Saint Andreas, Arkadia
Anchusa cespitosa *	Boraginaceae	whole plant	Lefka ori mountain
Anchusa cretica	Boraginaceae	aerial parts	Grammenochoria, Ioannina
Anthemis orientalis	Compositae	aerial parts	Parnonas mountain
Anthemis rigida subsp. *rigida*	Compositae	whole plant	Amnissos, Ierakleio, Crete
Arctium minus	Compositae	aerial parts	Athos mountain
Armeria canescens	Plumbaginaceae	flowering aerial parts	Tzoumerka mountain, northern Greece
Armeria canescens	Plumbaginaceae	whole plant	Ochi mountain, southern Evia

Artemisia absinthium	Compositae	aerial parts	Attiki
Artemisia arborescens	Compositae	aerial parts	Megala Chorafia, Chania, Crete
Arum idaeum *	Araceae	tubers	Lefka ori mountain, western Crete, Chania
Asperula boissieri *	Rubiaceae	whole plant	Gkiona mountain, central Greece
Asperula lutea *	Rubiaceae	aerial parts	Parnassos mountain
Asperula oetae *	Rubiaceae	aerial parts	Oiti mountain, central Greece
Asperula taygetea *	Rubiaceae	aerial parts	Parnonas mountain
Asphodeline liburnica	Liliaceae	aerial parts	Oiti mountain
Asphodeline lutea	Liliaceae	aerial parts	Idi mountain, Crete
Asphodeline lutea	Liliaceae	roots	Idi mountain, Crete
Astragalus angustifolius subsp. *angustifolius*	Leguminosae	flowering aerial parts	Lefka ori mountain
Astragalus angustifolius subsp. *angustifolius*	Leguminosae	flowering aerial parts	Parnassos mountain
Astragalus creticus subsp. *creticus* *	Leguminosae	flowering aerial parts	Idi mountain
Astragalus creticus subsp. *creticus* *	Leguminosae	roots	Idi mountain
Astragalus creticus subsp. *rumelicus*	Leguminosae	flowering aerial parts	Kyllini mountain
Astragalus creticus subsp. *rumelicus*	Leguminosae	roots	Idi mountain
Astragalus glycyphyllos	Leguminosae	flowering aerial parts	Koziakas mountain
Astragalus lacteus	Leguminosae	aerial parts, roots, fruits	Oiti mountain
Astragalus mayeri	Leguminosae	aerial parts with fruits	Vourinos mountain
Astragalus pubiflorus	Leguminosae	aerial parts, roots, fruits	Voion mountain
Astragalus sirinicus	Leguminosae	whole plant and roots	Smolikas mountain
Astragalus spruneri	Leguminosae	perennial aerial parts and annual parts without fruits	Immitos mountain
Astragalus spruneri	Leguminosae	fruits and seeds	Immitos mountain
Astragalus thracicus subsp. *parnassi*	Leguminosae	flowering aerial parts	Parnassos mountain
Astragalus thracicus subsp. *parnassi*	Leguminosae	roots	Parnassos mountain
Atractylis cancellata	Compositae	whole plant	Salamina island
Atractylis gummifera	Compositae	subaerial parts	Zaros, central Crete
Aubrieta deltoidea	Cruciferae	whole plant with flowers and fruits	Immitos mountain
Aubrieta scardica	Cruciferae	whole plant in fruiting	Tzoumerka mountain
Ballota pseudodictamnus subsp. *pseudodictamnus*	Lamiaceae	flowering aerial parts	Amnissos, Ierakleio, Crete
Bellis longifolia *	Compositae	aerial parts	Idi mountain, Crete
Berberis cretica	Berberidaceae	roots	Idi mountain, central Greece
Berberis cretica	Berberidaceae	aerial parts	Idi mountain, central Greece
Beta vulgaris subsp. *maritima*	Amaranthaceae	aerial parts	foothills of Immitos mountain, Attiki
Bituminaria bituminosa	Leguminosae	aerial parts	Leuka mountain, Chania, western Crete

Bornmuellera baldaccii subsp. *baldaccii*	Cruciferae	aerial parts in fruiting	Smolikas mountain
Bornmuellera tymphaea *	Cruciferae	aerial with fruits	Vourinos mountain
Brassica oleracea	Brassicaceae	aerial parts	Pateras mountain
Brassica oleracea var. botrytis	Brassicaceae	aerial parts	cultivated
Bryonia cretica subsp. *cretica*	Cucurbitaceae	aerial parts (stems, leaves and fruits)	Kokkini Chani, central Crete, Irakleio
Bryonia cretica subsp. *cretica*	Cucurbitaceae	underground parts-tubers	Kokkini Chani, central Crete, Irakleio
Bupleurum fruticosum	Umbelliferae	stems with leaves	Parnonas mountain
Buxus sempervirens	Buxaceae	aerial parts in fruiting	Smolikas mountain, northern Creece
Cakile maritima	Cruciferae	aerial with fruits	beach Cheronisi, Saint Andreas, Arkadia
Carlina corymbosa	Compositae	aerial parts	Idi mountain
Cedrus libani subsp. *brevifolia*	Pinaceae	leaves	Cyprus
Centaurea achaia *	Compositae	aerial parts	Pateras mountain
Centaurea affinis	Compositae	aerial parts	Koziakas mountain
Centaurea attica	Compositae	aerial parts	Penteli mountain
Centaurea epirota	Compositae	aerial parts	Tymfi mountain
Centaurea idaea *	Compositae	aerial parts	Idi mountain
Centaurea pannosa	Compositae	aerial parts	Athos mountain
Centaurea pelia *	Compositae	aerial parts	Parnassos mountain
Centaurea peucedanifolia *	Compositae	flowering aerial parts	Athos mountain
Centaurea raphanina subsp. *mixta* *	Compositae	rossete leaves	Parnonas mountain
Centaurea spinosa	Compositae	flowering aerial parts	Markopoulos, Attiki
Centranthus longiflorus subsp. *junceum*	Valerianaceae	aerial parts	Oiti mountain, central Greece
Cerastium candidissimum *	Caryophyllaceae	aerial parts	Ochi mountain
Ceratonia siliqua	Leguminosae	annual stems and leaves	Zaros, central Crete
Cerinthe major	Boraginaceae	aerial parts	Livadia, Viotia, central Greece
Chamaecytisus austriacus	Leguminosae	flowering-fruiting aerial parts	Smolikas mountain
Chamaecytisus creticus *	Leguminosae	aerial parts	Idi mountain
Chamaecytisus sagittale subsp. *sagittale*	Leguminosae	flowering aerial parts	Parnonas mountain
Chondrilla ramosissima *	Compositae	aerial parts	Lakonia
Chrysanthemum coronarium	Compositae	aerial parts	Saint Andreas, Lakonia
Chrysanthemum segetum	Compositae	aerial parts	Saint Andreas, Lakonia
Cichorium spinosum	Compositae	aerial parts	Leuka mountain, Chania, western Crete
Cionura erecta	Asclepiadaceae	aerial parts	foothills of Pateras mountain, Attiki
Cirsium heldreichii *	Compositae	aerial parts	Dirfis mountain
Colchicum parnassicum *	Liliaceae	bulbs	Parnassos mountain
Colchicum parnassicum *	Liliaceae	basal leaves	Parnassos mountain
Colutea arborescens	Leguminosae	aerial parts	Parnassos mountain

Convolvulus boissieri subsp. *parnassicus*	Convolvulaceae	aerial parts	Gkiona mountain, central Greece
Coridothymus capitatus	Lamiaceae	flowering aerial parts	Saronida, Attiki
Corydalis blanda subsp. *parnassica* *	Papaveraceae	aerial and underground parts	Parnassos mountain
Cotinus coggygria (Rhus cotinus)	Anacardiaceae	stems and leaves	Parnonas mountain, Arkadia
Crataegus pycnoloba *	Rosaceae	aerial parts	Killini mountain
Crepis incana *	Compositae	whole plant	Kyllini mountain
Crithmum maritimum	Umbelliferae	aerial parts	Saronida, Attiki
Crupina crupinastrum	Compositae	aerial parts	Parnonas mountain, Arkadia
Cupressus sempervirens (forma horizontalis)	Cupressaceae	branches and leaves	Idi mountain, Zaros canyon, Psiloritis, Crete
Cupressus sempervirens (forma horizontalis)	Cupressaceae	fruits	Idi mountain, Zaros canyon, Psiloritis, Crete
Cynoglossum columnae	Boraginaceae	aerial parts	Penteli mountain
Cynoglossum creticum	Boraginaceae	aerial parts with fruits	Zaros, central Crete
Cytinus hypocistis subsp. *clusii*	Cytinaceae	aerial parts	Zaros, central Crete
Cytinus hypocistis subsp. *clusii*	Cytinaceae	aerial parts	Leuka mountain, Chania, western Crete
Daphne laureotica	Thymelaeaceae	branches and leaves	Koziakas mountain-Trikala
Daphne oleoides	Thymelaeaceae	branches and leaves	Dirfis mountain, central Evia
Daphne sericea	Thymelaeaceae	branches and leaves	Leuka mountain, Chania, western Crete
Daphne sericea	Thymelaeaceae	branches and leaves	Leuka mountain, Chania, western Crete
Daucus carota	Umbelliferae	aerial parts	Zaros, central Crete
Dianthus haematocalyx subsp. *pindicola*	Caryophyllaceae	whole plant	Smolikas mountain
Dianthus haematocalyx subsp. *ventricosus* *	Caryophyllaceae	aerial parts (annual and perennial)	Oiti mountain
Dianthus serratifolius subsp. *serratifolius* *	Caryophyllaceae	aerial parts (annual and perennial)	Immitos mountain
Digitalis ferruginea	Scrophulariaceae	aerial parts	Xirovouni mountain-Evia
Digitalis ferruginea	Scrophulariaceae	roots	Xirovouni mountain-Evia
Dittrichia viscosa	Compositae	aerial parts	University campus, Zografou, Attiki
Doronicum columnae	Compositae	whole plant	Dirfis mountain
Dracunculus vulgaris	Araceae	tubers	foothills of Immitos mountain, Attiki
Dracunculus vulgaris	Araceae	aerial parts	foothills of Immitos mountain, Attiki
Drypis spinosa	Caryophyllaceae	aerial parts	Dirfis mountain
Drypis spinosa	Caryophyllaceae	aerial parts	Taygetos mountain
Ebenus cretica *	Leguminosae	aerial parts	Crete island

Ebenus sibthorpii *	Leguminosae	flowering aerial parts	Pateras mountain
Ecballium elaterium	Cucurbitaceae	aerial parts	Zaros, central Crete
Echinophora tenuifolia subsp. *sibthorpiana*	Umbelliferae	aerial parts	Stauromenos, Irakleio, central Crete
Echinops spinosissimus	Compositae	flowering aerial parts	Zaros, central Crete
Echium angustifolium	Boraginaceae	flowering aerial parts	Salamina island
Edraianthus graminifolius	Campanulaceae	whole plant	Giona mountain, central Greece
Epilobium angustifolium	Onagraceae	aerial parts	Pieria mountain, Pieria
Epilobium dodonaei	Onagraceae	aerial parts	Pieria mountain, Pieria
Epilobium hirsutum	Onagraceae	aerial parts	Zaros, central Crete
Epilobium parviflorum	Onagraceae	aerial parts	Parnonas mountain, Arkadia
Erodium moschatum	Onagraceae	aerial parts	Zaros, central Crete
Eruca sativa or *vesicaria*	Cruciferae	aerial parts	Gouves, Irakleio, central Crete
Eryngium amethystinum	Umbelliferae	aerial parts and roots	Parnonas mountain
Eryngium amorginum *	Umbelliferae	aerial parts	Kavousi, eastern Crete
Eryngium campestre	Umbelliferae	basal leaves and flowering stems	Zaros, central Greece
Eryngium creticum	Umbelliferae	flowering stems	between Vrises and Georgioupolis, western Crete
Eryngium maritimum	Umbelliferae	basal leaves and flowering stems	Georgioupolis beach, western Crete
Eryngium ternatum *	Umbelliferae	aerial parts	Crete island
Erysimum creticum *	Cruciferae	aerial parts	Tourloti, Lasithi, eastern Crete
Erysimum graecum *	Cruciferae	flowering aerial parts	Xirovouni mountain
Euphorbia acanthothamnos	Euphorbiaceae	aerial parts	Dafni, Attiki
Euphorbia deflexa	Euphorbiaceae	flowering aerial parts	Papigo mountain
Euphorbia glabriflora	Euphorbiaceae	aerial parts	mountain Vourinos, Kozani
Euphorbia myrsinites	Euphorbiaceae	flowering aerial parts	Ipiros
Foeniculum vulgare	Umbelliferae	aerial parts	University campus, Zografou, Attiki
Foeniculum vulgare	Umbelliferae	roots	University campus, Zografou, Attiki
Fraxinus ornus	Oleaceae	stems and leaves	University campus, Zografou, Attiki
Galega officinalis	Leguminosae	aerial parts	Koziakas mountain
Galium fruticosum	Rubiaceae	aerial parts	Canyon of Topolia, western Crete, Chania
Galium thymifolium *	Rubiaceae	aerial parts	Killini mountain, Korinthia
Galium verum subsp. *verum*	Rubiaceae	aerial parts	Killini mountain-Korinthia
Genista acanthoclada	Leguminosae	aerial parts	University campus, Zografou, Attiki

Genista depressa	Leguminosae	whole plant	Smolikas mountain
Genista hassertiana	Leguminosae	aerial parts	Vourinos mountain
Genista millii	Leguminosae	whole plant (roots and flowering aerial parts)	Oiti mountain
Geocaryum pindicolum *	Umbelliferae	aerial parts with fruits	Giona mountain
Geranium macrorrhizum	Geraniaceae	whole plant	mountain Dirfi, central Evia
Geranium subcaulescens	Geraniaceae	whole plant	mountain Timfi, Ioannina
Gladiolus italicus	Iridaceae	underground parts, tubers	Amorgos island
Gladiolus italicus	Iridaceae	aerial parts	Amorgos island
Globularia cordifolia	Globulariaceae	aerial parts	mountain Gkiona, central Greece
Glycyrrhiza glabra	Leguminosae	roots	Kefallonia
Helianthemum salicifolium	Cistaceae	aerial parts	Dirfi mountain, central Evia
Helleborus cyclophyllus	Ranunculaceae	roots	Dirfis mountain, Evia
Helleborus cyclophyllus	Ranunculaceae	aerial parts	Dirfis mountain-Evia
Helminthotheca (Picris) echioides	Compositae	aerial parts	Zaros, central Crete
Heracleum sphondylium	Umbelliferae	aerial parts	Voion mountain
Heracleum sphondylium subsp. *pyrenaicum*	Umbelliferae	aerial parts	Oiti mountain
Hesperis laciniata subsp. *laciniata*	Cruciferae	aerial parts	Dirfis mountain
Hippocrepis comosa	Leguminosae	flowering aerial parts	Oiti mountain
Hippocrepis emerus subsp. *emeroides*	Leguminosae	aerial parts	Immitos mountain
Hypericum cerastoides	Guttiferae	aerial parts in fruiting	Athos mountain
Hypericum empetrifolium subsp. *empetrifolium*	Guttiferae	aerial parts	Pateras mountain
Hypericum olympicum f. minus	Guttiferae	aerial parts and roots	Parnonas mountain
Hypericum perforatum	Guttiferae	aerial parts	Dirfis mountain
Hypericum rumeliacum subsp. *apollinis*	Guttiferae	aerial parts	Oiti mountain
Hypericum trichocaulon *	Guttiferae	aerial parts	Idi mountain
Hypericum triquetrifolium	Guttiferae	flowering aerial parts	Evia
Hyssopus officinalis	Lamiaceae	aerial parts	cultivated
Iberis sempervirens	Cruciferae	whole plant	Tzoumerka mountain
Inula candida subsp. *candida* *	Compositae	aerial parts (annual and perennial)	Kakopetros, Chania, western Crete
Inula candida subsp. *limonella* *	Compositae	aerial parts (annual and perennial)	Taygetos mountain
Inula crithmoides	Compositae	aerial parts (annual and perennial)	Saint Andreas, Cheronisi, Arkadia
Inula pseudolimonella *	Compositae	aerial parts (annual and perennial)	Dicti mountain
Inula verbascifolia subsp. *methanea* *	Compositae	flowering aerial parts	Immitos mountain
Inula verbascifolia subsp. *methanea* *	Compositae	aerial parts (annual and perennial)	foothills of Taygetos mountain
Iris attica	Iridaceae	whole plant	Salamina, Attiki
Iris germanica	Iridaceae	roots	Saint Andreas,

			Arkadia
Iris unguicularis subsp. *cretensis* *	Iridaceae	aerial parts	Zaros, Crete
Iris unguicularis subsp. *cretensis* *	Iridaceae	roots	Zaros, Crete
Isatis tinctoria	Cruciferae	aerial parts with flowers and fruits	foothills of Parnonas mountain
Juniperus communis subsp. *communis*	Cupressaceae	branches, leaves, fruits	Smolikas mountain
Juniperus drupacea	Cupressaceae	branches and leaves	Parnonas mountain, Arkadia
Juniperus drupacea	Cupressaceae	fruits	Parnonas mountain, Arkadia
Jurinea mollis	Compositae	aerial parts	Salamina island
Lamium garganicum subsp. *pictum* *	Lamiaceae	flowering aerial parts and the roots	Tzoumerka mountain
Laserpitium pseudomeum *	Umbelliferae	aerial parts (annual and perennial)	Giona mountain
Laserpitium siler subsp. *garganicum*	Umbelliferae	aerial parts	Koziakas mountain
Lavandula angustifolia	Leguminosae	aerial parts	cultivated
Lavandula stoechas	Lamiaceae	flowering aerial parts	eastern foothills, Parnonas mountain
Lembotropis nigricans subsp. *nigricans*	Leguminosae	flowering aerial parts	Zagorochoria, Ioannina
Leontodon hispidus	Compositae	whole plant	Tzoumerka mountain
Leontodon tuberosus	Compositae	whole plant	Zaros, central Crete
Linaria peloponnesiaca	Scrophulariaceae	whole plant	Gkiona mountain, central Greece
Lithodora hispidula subsp. *hispidula*	Boraginaceae	aerial parts	Asterousia mountain
Loranthus europaeus	Loranthaceae	whole plant	Parnonas mountain
Lupinus albus	Leguminosae	fruits and seeds	Kaisariani, Attiki
Lupinus albus	Leguminosae	aerial parts	Kaisariani, Attiki
Lutzia cretica *	Cruciferae	aerial parts, annual and perennial	eastern Crete
Lychnis coronaria	Caryophyllaceae	aerial parts	Dirfis mountain
Lysimachia serpyllifolia *	Primulaceae	whole plant	Parnassos mountain
Malabaila aurea	Umbelliferae	aerial parts	Parnonas mountain
Malva sylvestris	Malvaceae	aerial parts	cultivated
Marrubium peregrinum	Lamiaceae	aerial parts at the beginning of flowering	Vilia, Attiki
Marrubium thessalum	Lamiaceae	flowering aerial parts	Vourinos mountain
Marrubium velutinum subsp. *cylleneum* *	Lamiaceae	flowering aerial parts	Kyllini mountain
Marrubium velutinum subsp. *velutinum* *	Lamiaceae	flowering aerial parts	Oiti mountain
Marrubium vulgare	Lamiaceae	aerial parts	Timfi mountain
Matricaria recutita	Compositae	flowering aerial parts	Saronida, Attiki
Matthiola sinuata	Cruciferae	aerial parts	Vouliagmeni, Attiki
Medicago falcata	Leguminosae	flowering aerial parts	Kyllini mountain
Medicago marina	Leguminosae	roots	near Monemvasia, eastern Peloponissos
Medicago marina	Leguminosae	flowering aerial parts with fruits	near Monemvasia, eastern Peloponissos

Melilotus graecus *	Leguminosae	whole plant (with roots and fruits)	Parnassiada, Fokida
Melissa officinalis	Lamiaceae	aerial parts	cultivated
Mentha aquatica	Lamiaceae	aerial parts	Mainalo mountain
Mentha longifolia	Lamiaceae	flowering aerial parts	Zaros, central Crete
Mentha microphylla	Lamiaceae	aerial parts	cultivated
Mentha pulegium	Lamiaceae	aerial parts	Zaros, central Crete
Mentha spicata	Lamiaceae	aerial parts	cultivated
Micromeria graeca	Lamiaceae	flowering aerial parts	Pateras mountain
Micromeria juliana	Lamiaceae	aerial parts	Parnitha mountain
Micromeria nervosa	Lamiaceae	flowering aerial parts	eastern Crete (near Kavousi)
Minuartia juniperina subsp. *glandulifera*	Caryophyllaceae	aerial parts	Taygetos mountain
Minuartia stellata	Caryophyllaceae	aerial parts	Giona mountain
Morina persica	Morinaceae	aerial parts	Taygetos
Narcissus tazetta	Amaryllidaceae	tubers	Zaros, central Crete
Nepeta argolica subsp. *argolica* *	Lamiaceae	flowering aerial parts	Vilia, Attiki
Nepeta argolica subsp. *dirphya* *	Lamiaceae	flowering aerial parts, some with fruits	Dirfis mountain
Nepeta camphorata *	Lamiaceae	flowering aerial parts	Taygetos mountain
Nepeta melissifolia *	Lamiaceae	aerial parts	Crete island
Nepeta nuda	Lamiaceae	flowering aerial parts	Koziakas mountain
Nepeta orphanidea var. parnidea *	Lamiaceae	flowering aerial parts	Parnonas mountain
Nepeta parnassica	Lamiaceae	aerial parts just before flowering	Parnassos mountain
Nepeta spruneri	Lamiaceae	aerial parts	Tzoumerka mountain
Nepeta spruneri	Lamiaceae	perennial aerial parts	Timfi mountain
Odontites linkii subsp. *linkii* *	Scrophulariaceae	aerial parts	Parnonas mountain, Arkadia
Olea europaea	Oleaceae	leaves	Kalamata, Peloponissos
Onobrychis alba subsp. *laconica*	Leguminosae	annual flowering aerial parts	Parnonas mountain
Onobrychis alba subsp. *laconica*	Leguminosae	perennial aerial parts and roots	Parnonas mountain
Onobrychis alba subsp. *laconica*	Leguminosae	whole plant	Voion mountain
Onobrychis caput-galli	Leguminosae	aerial parts	Parnonas mountain
Onobrychis ebenoides *	Leguminosae	whole plant	Immitos mountain
Onobrychis peloponnesiaca *	Leguminosae	roots	Vlachiotis, Lakonia
Onobrychis peloponnesiaca *	Leguminosae	flowering aerial parts	Vlachiotis, Lakonia
Ononis pubescens	Leguminosae	whole plant (with flowers and fruits)	ancient Gortina, Crete
Ononis spinosa	Leguminosae	flowering aerial parts	Lefka ori mountain
Ononis viscosa	Leguminosae	whole plant with fruits	rim of Kaisariani stream, Attiki
Onosma elegantissima *	Boraginaceae	aerial parts (at the end of flowering)	Vourinos mountain
Onosma erecta subsp. *erecta* *	Boraginaceae	aerial parts	Lefka ori mountain

Onosma frutescens	Boraginaceae	aerial parts	Immitos mountain
Onosma pygmaea *	Boraginaceae	whole plant	Smolikas mountain
Orchis italica	Orchidaceae	leaves and flower stalks without inflorescences	Zaros, Crete
Orchis quadripunctata	Orchidaceae	leaves and flower stalks without inflorescences	Parnonas mountain-Arkadia
Origanum dictamnus *	Lamiaceae	flowering aerial parts	cultivated
Origanum majorana	Lamiaceae	Stems and inflorescences, no leaves	cultivated
Origanum microphyllum *	Lamiaceae	aerial parts	Crete island
Origanum onites	Lamiaceae	annual flowering aerial parts	Methana
Origanum vulgare subsp. *hirtum*	Lamiaceae	aerial parts	Parnonas mountain
Osyris alba	Santalaceae	aerial parts	foothills of Immitos mountain, Attiki
Otanthus maritimus	Compositae	aerial parts	southern Crete
Paeonia mascula subsp. *hellenica* *	Paeoniaceae	aerial parts in fruiting	Xirovouni mountain, Evia
Pallenis spinosa	Compositae	flowering aerial parts	Salamina island
Papaver rhoas	Papaveraceae	basal leaves (edible)	Zaros, Crete
Parietaria cretica	Urticaceae	aerial parts	Crete island
Parietaria diffusa	Urticaceae	aerial parts	Zaros, Crete
Paronychia albanica	Caryophyllaceae	aerial parts	Oiti mountain
Passiflora incarnata	Passifloraceae	aerial parts	cultivated
Petromarula pinnata *	Campanulaceae	aerial parts	Crete island
Peucedanum vourinense *	Umbelliferae	aerial parts	Vourinos mountain
Phlomis cretica *	Lamiaceae	flowering aerial parts	Zaros, central Crete
Phlomis fruticosa	Lamiaceae	flowering aerial parts	Fourfouras, Rethymno
Phlomis lanata *	Lamiaceae	flowering aerial parts	Zaros, central Crete
Phlomis samia	Lamiaceae	annual flowering aerial parts	Parnonas mountain
Phoenix theophrasti	Palmae	leaves and inflorescences of male trees	Raches
Phoenix theophrasti	Palmae	leaves of female trees	Preveli, Rethimno
Pimpinella tragium subsp. *tragium*	Umbelliferae	aerial parts (flowering annual and perennial)	Oiti mountain
Pinus heldreichii	Pinaceae	branches and leaves	Smolikas mountain, northern Greece
Plantago atrata subsp. *graeca*	Plantaginaceae	whole plant	Killini-Korinthia
Plantago coronopus subsp. *cummutata*	Plantaginaceae	whole plant	Zaros-Crete
Plantago holosteum	Plantaginaceae	whole plant	Killini mountain-Korinthia
Plantago lanceolata	Plantaginaceae	basal leaves	Zaros-Crete
Plantago major	Plantaginaceae	basal leaves	Parnonas mountain-Arkadia
Platanus orientalis	Platanaceae	platanus berries	Parnonas mountain-Prastos Arkadia
Polygonum aviculare	Polygonaceae	whole plant	Smolikas mountain, northern Greece
Polygonum tinctoria	Polygonaceae	whole plant (cultivated)	Panepistimiopolis

			Zografou
Portulaca oleracea	Portulacaceae	aerial parts	Zaros, Crete
Potentilla speciosa	Rosaceae	aerial parts	Oiti mountain
Prunella laciniata	Lamiaceae	flowering aerial parts	Tzoumerka mountain
Prunus spinosa	Rosaceae	stems, leaves and fruits	Athos mountain
Pseudorlaya pumila	Umbelliferae	whole plant at fruit development	beach Cheronisi, Saint Andreas, Arkadia
Psoralea bituminosa	Leguminosae	flowering aerial parts	Livadia
Pterocephalus perennis subsp. *perennis* *	Dipsacaceae	aerial parts (annual and perennial)	mountain Dirfi
Ptilostemon afer subsp. *afer*	Compositae	aerial parts	Dirfis mountain
Ptilostemon chamaepeuce	Compositae	aerial parts	foothills of Immitos mountain
Punica granatum	Punicaceae	pericarp	Zaros-Crete
Putoria calabrica	Rubiaceae	whole plant (aerial and roots)	Tzoumerka
Pyrus spinosa	Rosaceae	branches and leaves	Parnonas mountain
Quercus ithaburensis subsp. *macrolepis*	Leguminosae	stems and leaves	Rethymno, Crete
Raphanus sativus	Brassicaceae	bulbs	cultivated
Rhamnus alpina	Rhamnaceae	aerial parts	Voiou mountain-Kozani
Rhamnus alpina	Rhamnaceae	aerial parts	Gkiona mountain-central Greece
Rhamnus lycioides subsp. *graeca*	Rhamnaceae	aerial parts	Zaros-Crete
Rhamnus sibthorpiana *	Rhamnaceae	aerial parts	Koulochera mountain-Lakonia
Rosa damascena	Rosaceae	flowers	cultivated
Rosmarinus officinalis	Lamiaceae	aerial parts	near the Monastery of Kaisariani, Attiki
Rubia peregrina	Rubiaceae	flowering aerial parts	Parnonas mountain, Arkadia
Rubia tenuifolia	Rubiaceae	flowering aerial parts	Immitos mountain, central Greece
Rubia tinctorum	Rubiaceae	flowering aerial parts	Parnonas mountain, Arkadia, eastern Peloponnisos
Rubia tinctorum	Rubiaceae	radix	Parnonas mountain, Arkadia, eastern Peloponnisos
Ruta graveolens	Rutaceae	aerial parts in fruiting	Athos mountain
Salvia amplexicaulis	Lamiaceae	aerial parts at the end of flowering	Smolikas mountain
Salvia argentea	Lamiaceae	flowering aerial parts	Parnonas mountain
Salvia candidissima	Lamiaceae	perennial aerial parts and roots	Smolikas mountain
Salvia fruticosa	Lamiaceae	flowering aerial parts	Zaros, central Crete
Salvia officinalis	Lamiaceae	flowering aerial parts	between Kalpaki and Aristi, Ioannina
Salvia pomifera subsp. *calycina*	Lamiaceae	flowering aerial parts	Parnonas mountain
Salvia pomifera subsp. *pomifera*		flowering aerial parts	canyon of Topolia,

			Chania, Crete
Salvia ringens	Lamiaceae	aerial parts	Parnonas mountain
Salvia sclarea	Lamiaceae	flowering aerial parts	Timfi mountain
Salvia verbenaca	Lamiaceae	aerial parts	Sikelias hill, Attiki
Salvia viridis	Lamiaceae	aerial parts and roots	Parnonas mountain
Sambucus ebulus	Caprifoliaceae	branches and leaves	Ipiros
Sambucus nigra	Caprifoliaceae	branches and leaves	Athos mountain
Saponaria officinalis	Caryophyllaceae	flowering aerial parts	Athos mountain
Sarcopoterium spinosum	Rosaceae	aerial parts	Zaros, Crete
Satureja hortensis	Lamiaceae	aerial parts	cultivated
Satureja horvatii subsp. *macrophylla* *	Lamiaceae	flowering aerial parts	Tzoumerka mountain
Satureja montana subsp. *montana*	Lamiaceae	leaves, branches, roots	Smolikas mountain
Satureja parnassica subsp. *hellenica* *	Lamiaceae	flowering aerial parts	Parnitha mountain
Satureja parnassica subsp. *parnassica* *	Lamiaceae	flowering aerial parts	Parnonas mountain
Satureja spinosa	Lamiaceae	flowering aerial parts	eastern Crete
Satureja thymbra	Lamiaceae	flowering aerial parts	Ochi mountain
Satureja thymbra (montana ecotype)	Lamiaceae	aerial parts	Dicti mountain
Scabiosa crenata subsp. *crenata*	Dipsacaceae	whole plant in flowering	Tzoumerka mountain, northern Greece
Scabiosa crenata subsp. *crenata*	Dipsacaceae	whole plant in flowering	Smolikas mountain, northern Greece
Scandix pecten-veneris	Umbelliferae	rosette of basal leaves	Zaros, central Crete
Scolymus hispanicus	Compositae	aerial parts and part of the root	Zaros, central Crete
Scorzonera crocifolia *	Compositae	whole plant in flowering	Crete island
Scutellaria sieberi *	Lamiaceae	aerial parts	Crete island
Sedum album	Crassulaceae	aerial parts	Timfi mountain, Ioannina
Sedum sediforme	Crassulaceae	aerial parts	Peramos, Attiki
Selinum seilaifolium	Umbelliferae	aerial parts during flowering	Giona mountain
Senecio eubeus *	Compositae	flowering aerial parts	Xirovouni mountain
Senecio taygeteus *	Compositae	flowering aerial parts	Dirfis mountain
Senecio thapsoides	Compositae	flowering aerial parts	Oiti mountain
Sesamum indicum	Pedaliaceae	seeds	cultivated
Seseli rigidum	Umbelliferae	radix	Smolikas mountain
Sideritis clandestina subsp. *clandestina* *	Lamiaceae	aerial parts	Gaitanorachi, Parnonas mountain
Sideritis clandestina subsp. *peloponnesiaca* *	Lamiaceae	flowering aerial parts	Kyllini mountain
Sideritis curvidens	Lamiaceae	flowering aerial parts	Parnassos mountain
Sideritis euboea *	Lamiaceae	flowering aerial parts	Xirovouni mountain
Sideritis perfoliata subsp. *perfoliata*	Lamiaceae	aerial parts	Thessalia, central Greece
Sideritis raeseri subsp. *raeseri*	Lamiaceae	flowering aerial parts	Tymphi mountain
Sideritis romana subsp. *romana*	Lamiaceae	aerial parts	University campus, Zografou, Attiki
Sideritis scardica	Lamiaceae	aerial parts	cultivated

Sideritis syriaca subsp. *syriaca* *	Lamiaceae	flowering aerial parts	Idi mountain
Silene auriculata *	Caryophyllaceae	aerial parts	Kyllini mountain
Silene bupleuroides	Caryophyllaceae	aerial parts	Oiti mountain
Silene vulgaris	Caryophyllaceae	aerial parts	Zaros, central Crete
Silybum marianum	Compositae	aerial parts	cultivated
Sinapis alba	Cruciferae	aerial parts	foothills of Immitos mountain
Solanum melongena	Solanaceae	branches and leaves	Flaska, Santorini
Solanum melongena	Solanaceae	aerial parts (stems and leaves)	Leonidion, eastern Peloponnisos
Sonchus asper	Compositae	flowering aerial parts	Zaros, central Crete
Sorbus aria	Rosaceae	branches and leaves	Koziakas mountain
Sorbus umbellata	Rosaceae	branches and leaves	Koziakas mountain
Stachys chrysantha *	Lamiaceae	aerial parts	Parnonas mountain
Stachys germanica subsp. *heldreichii*	Lamiaceae	flowering aerial parts	Dirfi mountain
Stachys iva	Lamiaceae	flowering aerial parts	Vourinos mountain
Stachys leucoglossa	Lamiaceae	aerial parts	Athos mountain
Stachys scardica	Lamiaceae	flowering aerial parts	Vourinos mountain
Stachys spinosa *	Lamiaceae	aerial parts	Zaros, central Crete
Stachys spruneri *	Lamiaceae	flowering aerial parts	Pateras mountain
Stachys tymphaea	Lamiaceae	flowering aerial parts	Giona mountain
Staehelina petiolata *	Compositae	aerial parts	Crete island
Staehelina uniflosculosa	Compositae	aerial parts	Tzoumerka mountain
Styrax officinalis	Styracaceae	branches and leaves	Zaros, Crete
Tamus communis	Dioscoreaceae	underground parts	Zaros, central Crete
Tamus communis	Dioscoreaceae	aerial parts	Zaros, central Crete
Tanacetum parthenium	Compositae	flowering aerial parts	Parnonas mountain
Taxus baccata	Taxaceae	leaves	Parnonas mountain
Taxus baccata	Taxaceae	branches	Ioannina and Parnonas mountain
Taxus baccata	Taxaceae	leaves	Ioannina
Teline monspessulana	Leguminosae	aerial parts	foothills of Dirfis mountain
Tetragonolobus purpureus	Leguminosae	aerial parts	Zaros, central Crete
Teucrium capitatum	Lamiaceae	whole plant and roots	Xirovouni mountain
Teucrium chamaedrys subsp. *chamaedrys*	Lamiaceae	flowering aerial parts	Koziakas mountain
Teucrium divaricatum subsp. *divaricatum*	Lamiaceae	flowering aerial parts	Salamina island
Teucrium halacsyanum *	Lamiaceae	flowering aerial parts	Paliovouna mountain
Teucrium montanum subsp. *helianthemoiides* *	Lamiaceae	flowering aerial parts	Dirfi mountain
Teucrium montanum subsp. *montanum*	Lamiaceae	flowering aerial parts	Giona mountain
Thalictrum minus subsp. *olympicum*	Ranunculaceae	aerial parts	Gkiona mountain, central Greece
Thapsia garganica	Umbelliferae	aerial parts during flowering	Pines, eastern Crete
Thymelaea hirsuta *	Thymelaeaceae	aerial parts	foothills of Immitos, Attiki

Thymelaea tartonraira subsp. *argentea*	Thymelaeaceae	aerial parts	Kithaironas mountain
Thymus atticus	Lamiaceae	aerial parts	Immitos mountain
Thymus boissieri var. boissieri	Lamiaceae	flowering aerial parts	Tymfi mountain
Thymus leucospermus *	Lamiaceae	flowering aerial parts	Tymfi mountain
Thymus leucotrichus	Lamiaceae	flowering aerial parts	Giona mountain
Thymus longicaulis	Lamiaceae	flowering aerial parts	Kithaironas mountain
Thymus vulgaris	Lamiaceae	aerial parts	cultivated
Tragopogon porrifolius	Compositae	flowering aerial parts	Saronida, Attiki
Trifolium noricum	Leguminosae	whole plant (aerial and roots)	Giona mountain
Trifolium noricum	Leguminosae	whole plant in flowering	Tzoumerka mountain
Trifolium pratense	Leguminosae	aerial parts	Oiti mountain
Tussilago farfara	Compositae	leaves and roots	Tzoumerka mountain
Umbilicus horizontalis	Crassulaceae	aerial parts and roots	foothills of Immitos, Attiki
Urtica pilulifera	Urticaceae	whole plant	Attiki
Valeriana italica	Valerianaceae	tubers	Immitos mountain-Attiki
Valeriana italica	Valerianaceae	flowering aerial parts	Immitos mountain, Attiki
Valeriana tuberosa	Valerianaceae	tubers and flowering aerial parts	Parnassos mountain, central Greecce
Veratrum album	Liliaceae	flowering aerial parts and roots	Koziakas mountain, Trikala
Verbascum acaule *	Scrophulariaceae	whole plant	Taygetos mountain-Lakonia
Verbascum arcturus *	Scrophulariaceae	annual aerial parts	Chania, western Crete
Verbascum arcturus *	Scrophulariaceae	perennial aerial parts (woody)	Chania, western Crete
Verbascum daenzeri *	Scrophulariaceae	aerial parts	Parnonas mountain, Arkadia
Verbascum epixanthinum var. epixanthinum *	Scrophulariaceae	aerial parts	Gkiona mountain, central Greece
Verbascum macrurum	Scrophulariaceae	aerial parts	central Crete
Verbascum undulatum	Scrophulariaceae	aerial parts	Attiki
Verbena officinalis	Verbenaceae	aerial parts and roots	Zaros, Crete
Vicia faba	Leguminosae	aerial parts after the maturation of the fruits (cultivated)	Zaros, central Crete
Vincetoxicum creticum *	Asclepiadaceae	aerial parts and perennial bases (no roots)	mountain Dikti, eastern Crete
Viscum album (on Abies cephalonica)	Loranthaceae	whole plant	Parnonas mountain
Vitex agnus-castus	Verbenaceae	fruits	foothills of Parnonas mountain
Vitex agnus-castus	Verbenaceae	branches and leaves	foothills of Parnonas mountain, Arkadia

Supplementary Table 2. Initial evaluation of plant extracts.

	Plant species	Extraction solvent	DPPH scavenging (% at 200 µg/mL)	IC_{50} (µg/ml)	Tyrosinase inhibition (% at 100 µg/mL)	IC_{50} (µg/ml)	Highest non-cytotoxic concentration (µg/mL)	IC_{50} (µg/ml)	DCF basal	DCF stimulated
**1**	*Acantholimon androsaceum*	EtOAc	12.09		10.69					
**2**	*Acantholimon androsaceum*	MeOH	95.56	25.68	24.59		0.8	59.03	96	>100
**3**	*Acanthus spinosus*	EtOAc	9.95		47.90	87.23				
**4**	*Acanthus spinosus*	MeOH	15.28		8.41					
**5**	*Achillea abrotanoides*	EtOAc	7.82		4.92					
**6**	*Achillea abrotanoides*	MeOH	31.91		13.23					
**7**	*Achillea absinthoides*	EtOAc	10.65		16.76					
**8**	*Achillea absinthoides*	MeOH	38.84		17.61					
**9**	*Achillea cretica*	EtOAc	9.36		15.20					
**10**	*Achillea cretica*	MeOH	13.17		12.54					
**11**	*Achillea fraasii*	EtOAc	9.29		9.75					
**12**	*Achillea fraasii*	MeOH	33.88		16.10					
**13**	*Achillea maritima*	EtOAc	15.44		12.65					
**14**	*Achillea maritima*	MeOH	25.83		20.54					
**15**	*Achillea millefolium*	EtOAc	59.29		22.46		100	>100	59	49
**16**	*Achillea millefolium*	MeOH	52.29		28.65		100	>100	30	30
**17**	*Achillea pindicola* subsp. *intergifolia*	EtOAc	6.08		-1.57					
**18**	*Achillea pindicola* subsp. *intergifolia*	MeOH	49.43		20.59					
**19**	*Achillea taygetea*	EtOAc	12.96		16.85					
**20**	*Achillea taygetea*	MeOH	48.17		14.72					

**21**	*Achillea umbellata*	EtOAc	7.18		8.59						
**22**	*Achillea umbellata*	MeOH	57.53		12.61		4	>100	61	57	
**23**	*Acinos alpinus* subsp. *alpinus*	EtOAc	24.12		-2.62						
**24**	*Acinos alpinus* subsp. *alpinus*	MeOH	60.99		0.38		100	>100	72	86	
**25**	*Acinos alpinus* subsp. *meridionalis*	EtOAc	20.83		4.13						
**26**	*Acinos alpinus* subsp. *meridionalis*	MeOH	47.48		8.88						
**27**	*Acinos suaveolens*	EtOAc	9.37		10.58						
**28**	*Acinos suaveolens*	MeOH	26.05		8.30						
**29**	*Agrimonia eupatoria*	EtOAc	17.90		31.08						
**30**	*Agrimonia eupatoria*	MeOH	94.67	28.62	20.01		20	>100	59	56	
**31**	*Ajuga orientalis*	EtOAc	12.68		20.60						
**32**	*Ajuga orientalis*	MeOH	20.46		9.51						
**33**	*Alkana pindicola* subsp. *pindicola*	EtOAc	-1.68		23.88						
**34**	*Alkana pindicola* subsp. *pindicola*	MeOH	15.44		26.26						
**35**	*Allium ampeloprasum*	EtOAc	5.37		10.16						
**36**	*Allium ampeloprasum*	MeOH	5.95		6.28						
**37**	*Alyssum saxatile*	EtOAc	2.84		20.68						
**38**	*Alyssum saxatile*	MeOH	14.65		7.93						
**39**	*Alyssum smolikanum*	EtOAc	3.91		19.26						
**40**	*Alyssum smolikanum*	MeOH	13.68		11.46						
**41**	*Alyssum taygeteum*	EtOAc	6.01		7.24						
**42**	*Alyssum taygeteum*	MeOH	12.94		5.94						

**43**	*Amelanchier ovalis*	EtOAc	44.95		-4.77				
**44**	*Amelanchier ovalis*	MeOH	94.57	27.32	-5.45	20	>100	46	87
**45**	*Amelanchier parviflora* subsp. *chelmea*	EtOAc	41.77		3.19				
**46**	*Amelanchier parviflora* subsp. *chelmea*	MeOH	92.16	30.51	-4.99	0.8	>100	93	>100
**47**	*Anagyris foetida*	EtOAc	1.56		13.34				
**48**	*Anagyris foetida*	MeOH	12.71		12.21				
**49**	*Anchusa cespitosa*	EtOAc	19.39		22.40				
**50**	*Anchusa cespitosa*	MeOH	91.13	23.95	8.76	0.8	6.45	>100	95
**51**	*Anchusa cretica*	EtOAc	7.24		20.08				
**52**	*Anchusa cretica*	MeOH	58.21		14.37	20	>100	82	>100
**53**	*Anthemis orientalis*	EtOAc	27.37		31.40				
**54**	*Anthemis orientalis*	MeOH	53.17		26.15	100	>100	73	90
**55**	*Anthemis rigida* subsp. *rigida*	EtOAc	10.68		30.11				
**56**	*Anthemis rigida* subsp. *rigida*	MeOH	22.55		27.88				
**57**	*Arctium minus*	EtOAc	6.89		14.09				
**58**	*Arctium minus*	MeOH	28.10		23.57				
**59**	*Armeria canescens*	EtOAc	46.70		-2.99				
**60**	*Armeria canescens*	MeOH	58.66		10.05	4	>100	97	87
**61**	*Armeria canescens*	EtOAc	42.47		7.34				
**62**	*Armeria canescens*	MeOH	42.27		14.53				
**63**	*Artemisia absinthium*	EtOAc	56.31		34.05	0.8	84.66	85	>100
**64**	*Artemisia absinthium*	MeOH	55.32		39.60	100	>100	31	37
**65**	*Artemisia arborescens*	EtOAc	6.58		14.69				
**66**	*Artemisia arborescens*	MeOH	32.75		21.85				

**67**	*Arum idaeum*	EtOAc	10.72	20.12
**68**	*Arum idaeum*	MeOH	11.91	17.47
**69**	*Asperula boissieri*	EtOAc	9.03	32.32
**70**	*Asperula boissieri*	MeOH	20.71	10.51
**71**	*Asperula lutea*	EtOAc	4.40	13.33
**72**	*Asperula lutea*	MeOH	19.12	8.44
**73**	*Asperula oetae*	EtOAc	10.38	14.58
**74**	*Asperula oetae*	MeOH	15.38	-1.82
**75**	*Asperula taygetea*	EtOAc	3.98	33.36
**76**	*Asperula taygetea*	MeOH	14.27	1.03
**77**	*Asphodeline liburnica*	EtOAc	9.92	4.49
**78**	*Asphodeline liburnica*	MeOH	11.22	5.76
**79**	*Asphodeline lutea*	EtOAc	33.60	3.83
**80**	*Asphodeline lutea*	MeOH	32.27	4.50
**81**	*Asphodeline lutea*	EtOAc	33.18	6.58
**82**	*Asphodeline lutea*	MeOH	15.87	5.40
**83**	*Astragalus angustifolius* subsp. *angustifolius*	EtOAc	12.86	28.07
**84**	*Astragalus angustifolius* subsp. *angustifolius*	MeOH	15.17	18.37
**85**	*Astragalus angustifolius* subsp. *angustifolius*	EtOAc	9.84	27.04
**86**	*Astragalus angustifolius* subsp. *angustifolius*	MeOH	16.74	14.03

**87**	*Astragalus creticus* subsp. *creticus*	EtOAc	17.06	11.24
**88**	*Astragalus creticus* subsp. *creticus*	MeOH	15.65	11.63
**89**	*Astragalus creticus* subsp. *creticus*	EtOAc	13.39	12.14
**90**	*Astragalus creticus* subsp. *creticus*	MeOH	5.46	-3.49
**91**	*Astragalus creticus* subsp. *rumelicus*	EtOAc	26.79	20.14
**92**	*Astragalus creticus* subsp. *rumelicus*	MeOH	16.48	8.86
**93**	*Astragalus creticus* subsp. *rumelicus*	EtOAc	18.03	6.49
**94**	*Astragalus creticus* subsp. *rumelicus*	MeOH	9.65	-5.73
**95**	*Astragalus glycyphyllos*	EtOAc	3.39	26.68
**96**	*Astragalus glycyphyllos*	MeOH	8.37	23.95
**97**	*Astragalus lacteus*	EtOAc	22.67	17.88
**98**	*Astragalus lacteus*	MeOH	11.69	6.77
**99**	*Astragalus mayeri*	EtOAc	14.66	28.80
**100**	*Astragalus mayeri*	MeOH	12.67	26.15
**101**	*Astragalus pubiflorus*	EtOAc	9.55	27.20
**102**	*Astragalus pubiflorus*	MeOH	8.66	15.30
**103**	*Astragalus sirinicus*	EtOAc	17.87	24.00
**104**	*Astragalus sirinicus*	MeOH	11.65	13.94
**105**	*Astragalus spruneri*	EtOAc	5.99	21.96
**106**	*Astragalus spruneri*	MeOH	48.96	12.04
**107**	*Astragalus spruneri*	EtOAc	10.94	22.78
**108**	*Astragalus spruneri*	MeOH	44.59	16.13

**109**	*Astragalus thracicus* subsp. *parnassi*	EtOAc	6.59	25.34				
**110**	*Astragalus thracicus* subsp. *parnassi*	MeOH	11.98	21.61				
**111**	*Astragalus thracicus* subsp. *parnassi*	EtOAc	8.35	-1.36				
**112**	*Astragalus thracicus* subsp. *parnassi*	MeOH	7.27	-8.87				
**113**	*Atractylis cancellata*	EtOAc	5.12	29.40				
**114**	*Atractylis cancellata*	MeOH	14.71	8.06				
**115**	*Atractylis gummifera*	EtOAc	1.45	-6.66				
**116**	*Atractylis gummifera*	MeOH	56.14	-5.75	100	>100	>100	>100
**117**	*Aubrieta deltoidea*	EtOAc	30.07	18.82				
**118**	*Aubrieta deltoidea*	MeOH	37.42	14.53				
**119**	*Aubrieta scardica*	EtOAc	21.30	38.18				
**120**	*Aubrieta scardica*	MeOH	28.14	31.37				
**121**	*Ballota pseudodictamnus* subsp. *pseudodictamnus*	EtOAc	17.06	17.16				
**122**	*Ballota pseudodictamnus* subsp. *pseudodictamnus*	MeOH	51.32	11.34	100	>100	55	>100
**123**	*Bellis longifolia*	EtOAc	18.99	21.58				
**124**	*Bellis longifolia*	MeOH	45.12	24.56				

**125**	*Berberis cretica*	EtOAc	28.29		15.28					
**126**	*Berberis cretica*	MeOH	59.75		18.53		0.8	3.24	100	96
**127**	*Berberis cretica*	EtOAc	34.83		12.10					
**128**	*Berberis cretica*	MeOH	81.61	43.85	-0.64		20	84.06	>100	>100
**129**	*Beta vulgaris* subsp. *maritima*	EtOAc	18.00		22.80					
**130**	*Beta vulgaris* subsp. *maritima*	MeOH	16.78		20.70					
**131**	*Bituminaria bituminosa*	EtOAc	12.05		20.46					
**132**	*Bituminaria bituminosa*	MeOH	9.00		21.56					
**133**	*Bornmuellera baldachii* subsp. *baldachii*	EtOAc	4.12		13.07					
**134**	*Bornmuellera baldachii* subsp. *baldachii*	MeOH	22.33		0.14					
**135**	*Bornmuellera tymphaea*	EtOAc	5.78		0.49					
**136**	*Bornmuellera tymphaea*	MeOH	14.01		2.54					
**137**	*Brassica oleracea*	EtOAc	5.02		12.48					
**138**	*Brassica oleracea*	MeOH	6.53		15.84					
**139**	*Brassica oleracea* var. *botrytis*	EtOAc	5.13		15.65					
**140**	*Brassica oleracea* var. *botrytis*	MeOH	46.56		16.78					
**141**	*Bryonia cretica* subsp. *cretica*	EtOAc	9.29		11.62					
**142**	*Bryonia cretica* subsp. *cretica*	MeOH	3.65		10.81					
**143**	*Bryonia cretica* subsp. *cretica*	EtOAc	0.63		11.54					

**144**	*Bryonia cretica* subsp. *cretica*	MeOH	3.35	12.45				
**145**	*Bupleurum fruticosum*	EtOAc	15.97	14.84				
**146**	*Bupleurum fruticosum*	MeOH	38.67	12.09				
**147**	*Buxus sempervirens*	EtOAc	12.62	8.70				
**148**	*Buxus sempervirens*	MeOH	24.21	8.93				
**149**	*Cakile maritima*	EtOAc	7.63	16.38				
**150**	*Cakile maritima*	MeOH	14.14	9.50				
**151**	*Carlina corymbosa*	EtOAc	5.24	1.53				
**152**	*Carlina corymbosa*	MeOH	23.40	8.99				
**153**	*Cedrus libani* subsp. *brevifolia*	EtOAc	8.57	20.25				
**154**	*Cedrus libani* subsp. *brevifolia*	MeOH	48.03	31.06				
**155**	*Centaurea achaia*	EtOAc	8.78	4.34				
**156**	*Centaurea achaia*	MeOH	17.32	14.79				
**157**	*Centaurea affinis*	EtOAc	9.66	4.51				
**158**	*Centaurea affinis*	MeOH	21.23	15.42				
**159**	*Centaurea attica*	EtOAc	7.46	3.47				
**160**	*Centaurea attica*	MeOH	19.52	9.80				
**161**	*Centaurea epirota*	EtOAc	5.47	6.99				
**162**	*Centaurea epirota*	MeOH	22.26	14.09				
**163**	*Centaurea idaea*	EtOAc	17.79	21.98				
**164**	*Centaurea idaea*	MeOH	54.36	19.99	4	>100	>100	>100
**165**	*Centaurea pannosa*	EtOAc	5.54	14.44				
**166**	*Centaurea pannosa*	MeOH	19.22	20.21				
**167**	*Centaurea pelia*	EtOAc	11.16	7.94				
**168**	*Centaurea pelia*	MeOH	27.39	14.79				
**169**	*Centaurea peucedanifolia*	EtOAc	4.94	10.11				
**170**	*Centaurea peucedanifolia*	MeOH	18.65	15.09				

**171**	*Centaurea raphanina* subsp. *mixta*	EtOAc	10.93		10.37					
**172**	*Centaurea raphanina* subsp. *mixta*	MeOH	15.21		6.38					
**173**	*Centaurea spinosa*	EtOAc	9.15		14.38					
**174**	*Centaurea spinosa*	MeOH	21.61		17.49					
**175**	*Centranthus longiflorus* subsp. *junceum*	EtOAc	9.57		6.39					
**176**	*Centranthus longiflorus* subsp. *junceum*	MeOH	27.82		10.48					
**177**	*Cerastium candidissimum*	EtOAc	8.99		14.87					
**178**	*Cerastium candidissimum*	MeOH	11.34		11.09					
**179**	*Ceratonia siliqua*	EtOAc	96.25	22.48	-10.70		100	>100	62	93
**180**	*Ceratonia siliqua*	MeOH	96.14	21.67	-15.68		100	>100	>100	>100
**181**	*Cerinthe major*	EtOAc	3.56		16.45					
**182**	*Cerinthe major*	MeOH	58.09		16.13		0.16	0.65	100	93
**183**	*Chamaecytisus austriacus*	EtOAc	26.09		21.54					
**184**	*Chamaecytisus austriacus*	MeOH	28.58		45.77	56.64				
**185**	*Chamaecytisus creticus*	EtOAc	38.14		20.45					
**186**	*Chamaecytisus creticus*	MeOH	45.67		30.01					
**187**	*Chamaecytisus sagittale* subsp. *sagittale*	EtOAc	12.20		14.31					
**188**	*Chamaecytisus*	MeOH	28.59		3.02					

	sagittale subsp. *sagittale*			
**189**	*Chondrilla ramosissima*	EtOAc	1.64	0.64
**190**	*Chondrilla ramosissima*	MeOH	18.18	23.34
**191**	*Chrysanthemum coronarium*	EtOAc	9.85	14.66
**192**	*Chrysanthemum coronarium*	MeOH	37.59	9.68
**193**	*Chrysanthemum segetum*	EtOAc	9.26	21.75
**194**	*Chrysanthemum segetum*	MeOH	28.09	14.33
**195**	*Cichorium spinosum*	EtOAc	48.10	20.15
**196**	*Cichorium spinosum*	MeOH	21.33	29.58
**197**	*Cionura erecta*	EtOAc	8.89	20.69
**198**	*Cionura erecta*	MeOH	16.89	21.86
**199**	*Cirsium heldreichii*	EtOAc	9.47	9.31
**200**	*Cirsium heldreichii*	MeOH	44.22	15.46
**201**	*Colchicum parnassicum*	EtOAc	38.32	8.11
**202**	*Colchicum parnassicum*	MeOH	20.77	2.53
**203**	*Colchicum parnassicum*	EtOAc	44.50	15.01
**204**	*Colchicum parnassicum*	MeOH	41.13	13.23
**205**	*Colutea arborescens*	EtOAc	14.57	21.55
**206**	*Colutea arborescens*	MeOH	12.83	22.65
**207**	*Convolvulus boissieri* subsp.	EtOAc	26.24	19.96

	parnassicus									
**208**	*Convolvulus boissieri* subsp. *parnassicus*	MeOH	88.43	35.79	11.85		4	>100	35	75
**209**	*Coridothymus capitatus*	EtOAc	43.09		-23.37					
**210**	*Coridothymus capitatus*	MeOH	75.76		-7.95		20	75.25	>100	>100
**211**	*Corydalis blanda* subsp. *parnassica*	EtOAc	23.71		13.15					
**212**	*Corydalis blanda* subsp. *parnassica*	MeOH	29.43		15.47					
**213**	*Cotinus coggygria (Rhus cotinus)*	EtOAc	94.95	21.73	-11.44		20	70	48	75
**214**	*Cotinus coggygria (Rhus cotinus)*	MeOH	95.46	25.13	0.07		100	>100	42	89
**215**	*Crataegus pycnoloba*	EtOAc	42.50		27.65					
**216**	*Crataegus pycnoloba*	MeOH	95.09	27.17	4.09		20	64.27	44	>100
**217**	*Crepis incana*	EtOAc	1.58		11.26					
**218**	*Crepis incana*	MeOH	17.37		18.36					
**219**	*Crithmum maritimum*	EtOAc	12.36		22.58					
**220**	*Crithmum maritimum*	MeOH	55.66		36.97		20	>100	>100	90
**221**	*Crupina crupinastrum*	EtOAc	9.32		13.00					
**222**	*Crupina crupinastrum*	MeOH	12.33		19.45					
**223**	*Cupressus sempervirens (forma horizontalis)*	EtOAc	50.21		47.40	77.64	0.16	31.1	100	>100
**224**	*Cupressus*	MeOH	54.43		18.94		0.16	3.09	>100	97

	sempervirens (forma horizontalis)							
**225**	*Cupressus sempervirens (forma horizontalis)*	EtOAc	17.91	23.49				
**226**	*Cupressus sempervirens (forma horizontalis)*	MeOH	48.14	13.86				
**227**	*Cynoglossum columnae*	EtOAc	10.03	15.45				
**228**	*Cynoglossum columnae*	MeOH	25.89	19.47				
**229**	*Cynoglossum creticum*	EtOAc	10.13	22.41				
**230**	*Cynoglossum creticum*	MeOH	65.48	18.53	20	>100	92	>100
**231**	*Cytinus hypocistis* subsp. *clusii*	EtOAc	7.50	16.54				
**232**	*Cytinus hypocistis* subsp. *clusii*	MeOH	12.00	17.82				
**233**	*Cytinus hypocistis* subsp. *clusii*	EtOAc	6.32	19.87				
**234**	*Cytinus hypocistis* subsp. *clusii*	MeOH	0.98	17.55				
**235**	*Daphne laureotica*	EtOAc	28.67	13.99				
**236**	*Daphne laureotica*	MeOH	26.12	31.05				
**237**	*Daphne oleoides*	EtOAc	38.44	23.05				
**238**	*Daphne oleoides*	MeOH	42.51	13.31				
**239**	*Daphne sericea*	EtOAc	47.36	12.05				
**240**	*Daphne sericea*	MeOH	73.03	11.73	100	>100	29	50
**241**	*Daphne sericea*	EtOAc	45.00	15.41				
**242**	*Daphne sericea*	MeOH	49.00	16.87				
**243**	*Daucus carota*	EtOAc	5.17	23.32				
**244**	*Daucus carota*	MeOH	23.48	15.15				

**245**	*Dianthus haematocalyx* subsp. *pindicola*	EtOAc	11.44		13.88				
**246**	*Dianthus haematocalyx* subsp. *pindicola*	MeOH	15.66		9.97				
**247**	*Dianthus haematocalyx* subsp. *ventricosus*	EtOAc	12.94		10.20				
**248**	*Dianthus haematocalyx* subsp. *ventricosus*	MeOH	14.22		10.22				
**249**	*Dianthus serratifolius* subsp. *serratifolius*	EtOAc	11.77		13.02				
**250**	*Dianthus serratifolius* subsp. *serratifolius*	MeOH	19.19		13.23				
**251**	*Digitalis ferruginea*	EtOAc	20.43		2.20				
**252**	*Digitalis ferruginea*	MeOH	41.53		11.65				
**253**	*Digitalis ferruginea*	EtOAc	43.86		15.34				
**254**	*Digitalis ferruginea*	MeOH	92.47	29.32	19.83	4	>100	75	>100
**255**	*Dittrichia viscosa*	EtOAc	20.42		5.28				
**256**	*Dittrichia viscosa*	MeOH	71.48		9.09	100	>100	62	74
**257**	*Doronicum columnae*	EtOAc	9.26		15.89				
**258**	*Doronicum columnae*	MeOH	24.35		12.12				
**259**	*Dracunculus vulgaris*	EtOAc	6.06		17.72				
**260**	*Dracunculus vulgaris*	MeOH	18.99		19.39				
**261**	*Dracunculus vulgaris*	EtOAc	8.32		3.22				

**262**	*Dracunculus vulgaris*	MeOH	43.43		10.92				
**263**	*Drypis spinosa*	EtOAc	10.15		16.42				
**264**	*Drypis spinosa*	MeOH	13.53		27.90				
**265**	*Drypis spinosa*	EtOAc	13.91		20.18				
**266**	*Drypis spinosa*	MeOH	17.43		23.43				
**267**	*Ebenus cretica*	EtOAc	5.65		29.87				
**268**	*Ebenus cretica*	MeOH	49.67		28.46				
**269**	*Ebenus sibthorpii*	EtOAc	16.36		32.45				
**270**	*Ebenus sibthorpii*	MeOH	82.22	53.69	31.82	100	>100	48	>100
**271**	*Ecballium elaterium*	EtOAc	2.72		21.61				
**272**	*Ecballium elaterium*	MeOH	3.13		24.62				
**273**	*Echinophora tenuifolia* subsp. *sibthorpiana*	EtOAc	8.99		18.01				
**274**	*Echinophora tenuifolia* subsp. *sibthorpiana*	MeOH	20.60		12.90				
**275**	*Echinops spinosissimus*	EtOAc	21.55		15.15				
**276**	*Echinops spinosissimus*	MeOH	57.27		11.62	4	>100	>100	>100
**277**	*Echium angustifolium*	EtOAc	3.62		17.14				
**278**	*Echium angustifolium*	MeOH	30.06		28.63				
**279**	*Edraianthus graminifolius*	EtOAc	4.32		19.16				
**280**	*Edraianthus graminifolius*	MeOH	14.77		12.87				
**281**	*Epilobium angustifolium*	EtOAc	13.85		17.45				
**282**	*Epilobium angustifolium*	MeOH	95.12	47.2	19.12	20	71.9	56	>100

**283**	*Epilobium dodonaei*	EtOAc	47.56		27.45					
**284**	*Epilobium dodonaei*	MeOH	95.62	22.23	33.24		100	>100	>100	>100
**285**	*Epilobium hirsutum*	EtOAc	27.41		16.06					
**286**	*Epilobium hirsutum*	MeOH	95.54	37.14	28.89		20	83.96	43	>100
**287**	*Epilobium parviflorum*	EtOAc	14.14		20.70					
**288**	*Epilobium parviflorum*	MeOH	95.41	30.42	28.41		100	>100	>100	>100
**289**	*Erodium moschatum*	EtOAc	16.03		14.78					
**290**	*Erodium moschatum*	MeOH	88.32	36.05	23.90		0.8	12.55	92	95
**291**	*Eruca sativa* or *vesicaria*	EtOAc	3.51		16.69					
**292**	*Eruca sativa* or *vesicaria*	MeOH	8.61		1.96					
**293**	*Eryngium amethystinum*	EtOAc	10.00		18.80					
**294**	*Eryngium amethystinum*	MeOH	29.59		18.00					
**295**	*Eryngium amorginum*	EtOAc	9.74		56.13	79.83				
**296**	*Eryngium amorginum*	MeOH	15.60		13.34					
**297**	*Eryngium campestre*	EtOAc	12.31		18.59					
**298**	*Eryngium campestre*	MeOH	58.20		19.04		0.8	2.12	>100	>100
**299**	*Eryngium creticum*	EtOAc	10.77		14.42					
**300**	*Eryngium creticum*	MeOH	19.23		12.31					
**301**	*Eryngium maritimum*	EtOAc	7.90		17.95					
**302**	*Eryngium maritimum*	MeOH	11.15		15.33					

**303**	*Eryngium ternatum*	EtOAc	18.23		22.89				
**304**	*Eryngium ternatum*	MeOH	43.00		23.46				
**305**	*Erysimum creticum*	EtOAc	4.27		15.39				
**306**	*Erysimum creticum*	MeOH	14.15		4.44				
**307**	*Erysimum graecum*	EtOAc	1.18		27.47				
**308**	*Erysimum graecum*	MeOH	5.70		15.00				
**309**	*Euphorbia acanthothamnos*	EtOAc	38.10		-1.54				
**310**	*Euphorbia acanthothamnos*	MeOH	95.58	26.05	29.92	100	>100	46	72
**311**	*Euphorbia deflexa*	EtOAc	13.40		11.21				
**312**	*Euphorbia deflexa*	MeOH	90.46	33.35	13.41	100	>100	66	77
**313**	*Euphorbia glabriflora*	EtOAc	19.03		10.99				
**314**	*Euphorbia glabriflora*	MeOH	95.39	26.68	37.46	100	>100	51	83
**315**	*Euphorbia myrsinites*	EtOAc	5.35		13.85				
**316**	*Euphorbia myrsinites*	MeOH	31.01		10.89				
**317**	*Foeniculum vulgare*	EtOAc	3.56		24.27				
**318**	*Foeniculum vulgare*	MeOH	10.81		14.11				
**319**	*Foeniculum vulgare*	EtOAc	13.66		22.33				
**320**	*Foeniculum vulgare*	MeOH	13.72		8.75				
**321**	*Fraxinus ornus*	EtOAc	43.06		22.32				
**322**	*Fraxinus ornus*	MeOH	41.77		10.32				
**323**	*Galega officinalis*	EtOAc	5.33		20.26				
**324**	*Galega officinalis*	MeOH	9.47		21.59				
**325**	*Galium fruticosum*	EtOAc	7.65		10.39				
**326**	*Galium fruticosum*	MeOH	55.41		11.84	20	61.07	>100	>100
**327**	*Galium thymifolium*	EtOAc	11.94		11.69				
**328**	*Galium thymifolium*	MeOH	33.48		12.92				

**329**	*Galium verum* subsp. *verum*	EtOAc	15.49		-7.87					
**330**	*Galium verum* subsp. *verum*	MeOH	47.41		5.04					
**331**	*Genista acanthoclada*	EtOAc	31.07		9.51					
**332**	*Genista acanthoclada*	MeOH	28.42		4.92					
**333**	*Genista depressa*	EtOAc	44.22		53.23	92.2				
**334**	*Genista depressa*	MeOH	34.51		16.46					
**335**	*Genista hassertiana*	EtOAc	41.21		19.22					
**336**	*Genista hassertiana*	MeOH	30.16		6.76					
**337**	*Genista millii*	EtOAc	30.26		-1.79					
**338**	*Genista millii*	MeOH	22.83		-14.53					
**339**	*Geocaryum pindicolum*	EtOAc	0.65		6.80					
**340**	*Geocaryum pindicolum*	MeOH	15.73		30.08					
**341**	*Geranium macrorrhizum*	EtOAc	94.66	25.64	29.07		100	>100	49	74
**342**	*Geranium macrorrhizum*	MeOH	94.57	17.54	54.69	72.88	20	72.88	40	93
**343**	*Geranium subcaulescens*	EtOAc	28.92		28.74					
**344**	*Geranium subcaulescens*	MeOH	95.13	28.16	30.92		100	>100	59	>100
**345**	*Gladiolus italicus*	EtOAc	20.17		12.97					
**346**	*Gladiolus italicus*	MeOH	33.86		11.55					
**347**	*Gladiolus italicus*	EtOAc	16.40		19.06					
**348**	*Gladiolus italicus*	MeOH	21.49		10.69					
**349**	*Globularia cordifolia*	EtOAc	80.41	55.97	18.34		20	>100	84	>100
**350**	*Globularia cordifolia*	MeOH	88.89	45.58	18.28		4	>100	>100	>100
**351**	*Glycyrrhiza glabra*	EtOAc	49.96		81.26	30.52				
**352**	*Glycyrrhiza glabra*	MeOH	96.33	35.7	91.78	29.53	4	29.53	73	97

**353**	*Helianthemum salicifolium*	EtOAc	15.33		13.99					
**354**	*Helianthemum salicifolium*	MeOH	85.23	49.88	16.43		20	56.42	58	60
**355**	*Helleborus cyclophyllus*	EtOAc	0.35		19.61					
**356**	*Helleborus cyclophyllus*	MeOH	1.81		2.49					
**357**	*Helleborus cyclophyllus*	EtOAc	6.76		16.88					
**358**	*Helleborus cyclophyllus*	MeOH	30.48		15.08					
**359**	*Helminthotheca (Picris) echioides*	EtOAc	9.90		8.42					
**360**	*Helminthotheca (Picris) echioides*	MeOH	28.25		7.20					
**361**	*Heracleum sphondylium*	EtOAc	4.70		29.83					
**362**	*Heracleum sphondylium*	MeOH	5.27		18.48					
**363**	*Heracleum sphondylium* subsp. *pyrenaicum*	EtOAc	10.19		43.35	96.48				
**364**	*Heracleum sphondylium* subsp. *pyrenaicum*	MeOH	7.01		19.16					
**365**	*Hesperis laciniata* subsp. *laciniata*	EtOAc	13.67		6.21					
**366**	*Hesperis laciniata* subsp. *laciniata*	MeOH	30.23		5.20					
**367**	*Hippocrepis comosa*	EtOAc	48.69		54.30	89.15				
**368**	*Hippocrepis comosa*	MeOH	49.51		54.53	76.52				
**369**	*Hippocrepis emerus* subsp. *emeroides*	EtOAc	40.31		35.78					

**370**	*Hippocrepis emerus* subsp. *emeroides*	MeOH	49.00		36.89					
**371**	*Hypericum cerastoides*	EtOAc	18.25		13.07					
**372**	*Hypericum cerastoides*	MeOH	70.34		13.62		100	>100	77	82
**373**	*Hypericum empetrifolium* subsp. *empetrifolium*	EtOAc	38.72		-13.15					
**374**	*Hypericum empetrifolium* subsp. *empetrifolium*	MeOH	92.11	29.91	5.35		20	61.44	96	91
**375**	*Hypericum olympicum f. minus*	EtOAc	22.16		22.30					
**376**	*Hypericum olympicum f. minus*	MeOH	76.56		19.54		100	>100	74	77
**377**	*Hypericum perforatum*	EtOAc	68.74		8.26		0.8	8.09	92	74
**378**	*Hypericum perforatum*	MeOH	85.91	47.7	45.43	88.51	0.8	7.88	32	68
**379**	*Hypericum rumeliacum* subsp. *apollinis*	EtOAc	42.69		11.95					
**380**	*Hypericum rumeliacum* subsp. *apollinis*	MeOH	92.59	30.68	8.45		20	>100	85	>100
**381**	*Hypericum trichocaulon*	EtOAc	23.02		15.57					
**382**	*Hypericum trichocaulon*	MeOH	92.29	30.35	10.45		100	>100	51	>100
**383**	*Hypericum triquetrifolium*	EtOAc	17.70		5.40					

**384**	*Hypericum triquetrifolium*	MeOH	90.81	32.64	9.20		20	>100	89	>100
**385**	*Hyssopus officinalis*	EtOAc	69.85		27.93		20	52.58	96	71
**386**	*Hyssopus officinalis*	MeOH	83.23	50.16	46.20	94.05	20	>100	62	61
**387**	*Iberis sempervirens*	EtOAc	6.27		15.68					
**388**	*Iberis sempervirens*	MeOH	13.08		11.75					
**389**	*Inula candida* subsp. *candida*	EtOAc	7.74		7.35					
**390**	*Inula candida* subsp. *candida*	MeOH	52.16		12.56		100	>100	91	90
**391**	*Inula candida* subsp. *limonella*	EtOAc	9.50		14.17					
**392**	*Inula candida* subsp. *limonella*	MeOH	60.00		6.08		100	>100	81	95
**393**	*Inula crithmoides*	EtOAc	10.43		24.37					
**394**	*Inula crithmoides*	MeOH	16.57		23.22					
**395**	*Inula pseudolimonella*	EtOAc	21.13		6.71					
**396**	*Inula pseudolimonella*	MeOH	50.72		9.83		0.8	>100	>100	>100
**397**	*Inula verbascifolia* subsp. *methanea*	EtOAc	8.16		8.29					
**398**	*Inula verbascifolia* subsp. *methanea*	MeOH	27.49		7.46					
**399**	*Inula verbascifolia* subsp. *methanea*	EtOAc	11.57		14.63					
**400**	*Inula verbascifolia* subsp. *methanea*	MeOH	34.98		16.51					
**401**	*Iris attica*	EtOAc	16.81		1.01					
**402**	*Iris attica*	MeOH	19.61		-6.87					
**403**	*Iris germanica*	EtOAc	11.04		2.36					
**404**	*Iris germanica*	MeOH	13.34		2.47					
**405**	*Iris unguicularis* subsp. *cretensis*	EtOAc	14.31		9.30					

**406**	*Iris unguicularis* subsp. *cretensis*	MeOH	70.33		9.55		0.8	8.32	>100	96
**407**	*Iris unguicularis* subsp. *cretensis*	EtOAc	71.61		3.46		100	>100	93	>100
**408**	*Iris unguicularis* subsp. *cretensis*	MeOH	70.33		-13.63		20	>100	>100	>100
**409**	*Isatis tinctoria*	EtOAc	4.08		16.51					
**410**	*Isatis tinctoria*	MeOH	11.70		2.67					
**411**	*Juniperus communis* subsp. *communis*	EtOAc	25.29		7.23					
**412**	*Juniperus communis* subsp. *communis*	MeOH	79.86		18.16		0.8	44.7	100	>100
**413**	*Juniperus drupacea*	EtOAc	9.05		24.97					
**414**	*Juniperus drupacea*	MeOH	88.40	45.45	19.42		0.8	37.82	>100	>100
**415**	*Juniperus drupacea*	EtOAc	46.53		22.18					
**416**	*Juniperus drupacea*	MeOH	94.79	37.63	33.68		0.16	0.67	>100	>100
**417**	*Jurinea molis*	EtOAc	7.52		10.55					
**418**	*Jurinea molis*	MeOH	25.97		17.51					
**419**	*Lamium garganicum* subsp. *pictum*	EtOAc	13.87		5.38					
**420**	*Lamium garganicum* subsp. *pictum*	MeOH	38.23		6.52					
**421**	*Laserpitium pseudomeum*	EtOAc	3.47		12.36					
**422**	*Laserpitium pseudomeum*	MeOH	13.62		9.04					
**423**	*Laserpitium siler* subsp. *garganicum*	EtOAc	2.41		22.65					
**424**	*Laserpitium siler* subsp. *garganicum*	MeOH	24.03		14.84					

**425**	*Lavandula angustifolia*	EtOAc	9.84		25.48					
**426**	*Lavandula angustifolia*	MeOH	12.32		29.45					
**427**	*Lavandula stoechas*	EtOAc	10.11		20.64					
**428**	*Lavandula stoechas*	MeOH	86.38	48.76	4.50		4	16.21	>100	95
**429**	*Lembotropis nigricans* subsp. *nigricans*	EtOAc	42.09		24.87					
**430**	*Lembotropis nigricans* subsp. *nigricans*	MeOH	90.32	42.79	17.02		100	>100	64	53
**431**	*Leontodon hispidus*	EtOAc	2.99		23.16					
**432**	*Leontodon hispidus*	MeOH	30.01		16.66					
**433**	*Leontodon tuberosus*	EtOAc	5.81		11.67					
**434**	*Leontodon tuberosus*	MeOH	54.00		11.38		100	>100	>100	>100
**435**	*Linaria peloponnesiaca*	EtOAc	4.82		5.20					
**436**	*Linaria peloponnesiaca*	MeOH	10.53		5.19					
**437**	*Lithodora hispidula* subsp. *hispidula*	EtOAc	22.65		19.36					
**438**	*Lithodora hispidula* subsp. *hispidula*	MeOH	88.04	45.22	17.21		100	>100	56	78
**439**	*Loranthus europaeus*	EtOAc	81.41	34.86	-18.11		100	>100	43	65
**440**	*Loranthus europaeus*	MeOH	94.29	28.01	-10.55		100	>100	48	91
**441**	*Lupinus albus*	EtOAc	3.81		4.64					
**442**	*Lupinus albus*	MeOH	7.12		2.09					
**443**	*Lupinus albus*	EtOAc	14.20		14.11					
**444**	*Lupinus albus*	MeOH	13.73		21.13					

**445**	*Lutzia cretica*	EtOAc	8.29	16.90				
**446**	*Lutzia cretica*	MeOH	13.72	6.56				
**447**	*Lychnis coronaria*	EtOAc	8.78	16.14				
**448**	*Lychnis coronaria*	MeOH	11.48	13.17				
**449**	*Lysimachia serpyllifolia*	EtOAc	20.54	12.76				
**450**	*Lysimachia serpyllifolia*	MeOH	44.43	9.11				
**451**	*Malabaila aurea*	EtOAc	15.11	33.68				
**452**	*Malabaila aurea*	MeOH	17.31	21.09				
**453**	*Malva sylvestris*	EtOAc	50.04	0.25	0.8	27.29	>100	89
**454**	*Malva sylvestris*	MeOH	50.61	1.26	4	9.53	99	90
**455**	*Marrubium peregrinum*	EtOAc	18.79	13.51				
**456**	*Marrubium peregrinum*	MeOH	28.04	6.49				
**457**	*Marrubium thessalum*	EtOAc	11.87	13.70				
**458**	*Marrubium thessalum*	MeOH	29.20	4.19				
**459**	*Marrubium velutinum* subsp. *cylleneum*	EtOAc	14.60	12.87				
**460**	*Marrubium velutinum* subsp. *cylleneum*	MeOH	27.64	1.37				
**461**	*Marrubium velutinum* subsp. *velutinum*	EtOAc	13.50	6.88				
**462**	*Marrubium velutinum* subsp. *velutinum*	MeOH	55.43	1.25	100	>100	>100	>100
**463**	*Marrubium vulgare*	EtOAc	18.47	19.52				
**464**	*Marrubium vulgare*	MeOH	33.33	27.16				

**465**	*Matricaria recutita*	EtOAc	13.22		19.16					
**466**	*Matricaria recutita*	MeOH	30.74		14.62					
**467**	*Matthiola sinuata*	EtOAc	9.11		22.21					
**468**	*Matthiola sinuata*	MeOH	10.55		11.28					
**469**	*Medicago falcata*	EtOAc	4.31		23.56					
**470**	*Medicago falcata*	MeOH	9.71		15.42					
**471**	*Medicago marina*	EtOAc	6.42		29.12					
**472**	*Medicago marina*	MeOH	0.29		19.79					
**473**	*Medicago marina*	EtOAc	2.37		20.09					
**474**	*Medicago marina*	MeOH	5.13		20.54					
**475**	*Melilotus graecus*	EtOAc	3.60		18.50					
**476**	*Melilotus graecus*	MeOH	17.37		35.15					
**477**	*Melissa officinalis*	EtOAc	58.91		15.23		4	10.92	57	63
**478**	*Melissa officinalis*	MeOH	90.17	33.03	44.18	78.23	20	57.19	59	44
**479**	*Mentha aquatica*	EtOAc	22.64		13.82					
**480**	*Mentha aquatica*	MeOH	75.81		10.76		100	>100	40	>100
**481**	*Mentha longifolia*	EtOAc	26.15		16.32					
**482**	*Mentha longifolia*	MeOH	67.06		14.60		100	>100	35	>100
**483**	*Mentha microphylla*	EtOAc	45.00		20.46					
**484**	*Mentha microphylla*	MeOH	48.00		28.06					
**485**	*Mentha pulegium*	EtOAc	32.86		43.35	88.9				
**486**	*Mentha pulegium*	MeOH	76.95		11.23		100	>100	64	>100
**487**	*Mentha spicata*	EtOAc	24.80		14.59					
**488**	*Mentha spicata*	MeOH	51.49		36.58		100	>100	51	75
**489**	*Micromeria graeca*	EtOAc	14.56		24.27					
**490**	*Micromeria graeca*	MeOH	87.70	46.63	13.41		20	67.05	55	82
**491**	*Micromeria juliana*	EtOAc	15.66		19.45					
**492**	*Micromeria juliana*	MeOH	40.83		29.46					
**493**	*Micromeria nervosa*	EtOAc	10.98		15.02					
**494**	*Micromeria nervosa*	MeOH	88.56	55.44	13.74		100	>100	63	95
**495**	*Minuartia juniperina* subsp. *glandulifera*	EtOAc	9.28		12.93					
**496**	*Minuartia*	MeOH	17.11		12.95					

	juniperina subsp. *glandulifera*							
**497**	*Minuartia stellata*	EtOAc	9.99	8.17				
**498**	*Minuartia stellata*	MeOH	15.53	13.24				
**499**	*Morina persica*	EtOAc	11.41	13.30				
**500**	*Morina persica*	MeOH	37.20	8.60				
**501**	*Narcissus tazetta*	EtOAc	8.08	15.07				
**502**	*Narcissus tazetta*	MeOH	19.73	16.92				
**503**	*Nepeta argolica* subsp. *argolica*	EtOAc	5.01	3.64				
**504**	*Nepeta argolica* subsp. *argolica*	MeOH	26.45	14.34				
**505**	*Nepeta argolica* subsp. *dirphya*	EtOAc	4.91	9.31				
**506**	*Nepeta argolica* subsp. *dirphya*	MeOH	29.46	16.81				
**507**	*Nepeta camphorata*	EtOAc	5.77	11.50				
**508**	*Nepeta camphorata*	MeOH	32.06	19.54				
**509**	*Nepeta melissifolia*	EtOAc	2.59	19.18				
**510**	*Nepeta melissifolia*	MeOH	34.98	25.08				
**511**	*Nepeta nuda*	EtOAc	3.65	7.28				
**512**	*Nepeta nuda*	MeOH	34.27	23.63				
**513**	*Nepeta orphanidea* var. *parnidea*	EtOAc	6.95	-0.55				
**514**	*Nepeta orphanidea* var. *parnidea*	MeOH	32.86	20.24				
**515**	*Nepeta parnassica*	EtOAc	5.15	14.61				
**516**	*Nepeta parnassica*	MeOH	18.53	22.81				
**517**	*Nepeta spruneri*	EtOAc	4.96	11.21				
**518**	*Nepeta spruneri*	MeOH	57.44	16.11	100	>100	26	72
**519**	*Nepeta spruneri*	EtOAc	23.98	17.31				
**520**	*Nepeta spruneri*	MeOH	42.97	23.11				
**521**	*Odontites linkii* subsp. *linkii*	EtOAc	12.28	29.55				

**522**	*Odontites linkii* subsp. *linkii*	MeOH	23.35	14.93	
**523**	*Olea europaea*	EtOAc	15.33	9.49	
**524**	*Olea europaea*	MeOH	45.03	-8.38	
**525**	*Onobrychis alba* subsp. *laconica*	EtOAc	6.60	24.98	
**526**	*Onobrychis alba* subsp. *laconica*	MeOH	42.19	14.04	
**527**	*Onobrychis alba* subsp. *laconica*	EtOAc	32.66	37.45	
**528**	*Onobrychis alba* subsp. *laconica*	MeOH	41.89	28.56	
**529**	*Onobrychis alba* subsp. *laconica*	EtOAc	40.71	42.74	76.52
**530**	*Onobrychis alba* subsp. *laconica*	MeOH	43.54	38.57	
**531**	*Onobrychis caput-galli*	EtOAc	0.17	10.56	
**532**	*Onobrychis caput-galli*	MeOH	25.67	18.45	
**533**	*Onobrychis ebenoides*	EtOAc	23.17	28.74	
**534**	*Onobrychis ebenoides*	MeOH	49.07	23.95	
**535**	*Onobrychis peloponnesiaca*	EtOAc	27.27	9.97	
**536**	*Onobrychis peloponnesiaca*	MeOH	43.12	15.51	
**537**	*Onobrychis peloponnesiaca*	EtOAc	41.02	42.35	78.49
**538**	*Onobrychis peloponnesiaca*	MeOH	42.17	33.66	
**539**	*Ononis pubescens*	EtOAc	13.45	12.67	
**540**	*Ononis pubescens*	MeOH	19.66	12.18	

**541**	*Ononis spinosa*	EtOAc	15.91		29.14					
**542**	*Ononis spinosa*	MeOH	24.21		25.48					
**543**	*Ononis viscosa*	EtOAc	8.38		23.93					
**544**	*Ononis viscosa*	MeOH	19.49		16.34					
**545**	*Onosma elegantissima*	EtOAc	5.01		20.77					
**546**	*Onosma elegantissima*	MeOH	74.21		17.77		100	>100	48	76
**547**	*Onosma erecta* subsp. *erecta*	EtOAc	10.16		16.70					
**548**	*Onosma erecta* subsp. *erecta*	MeOH	83.55	41.01	10.99		100	>100	69	>100
**549**	*Onosma frutescens*	EtOAc	7.19		17.07					
**550**	*Onosma frutescens*	MeOH	87.99	45.05	20.62		100	>100	56	74
**551**	*Onosma pygmaea*	EtOAc	26.77		22.86					
**552**	*Onosma pygmaea*	MeOH	90.89	41.06	18.98		100	>100	35	75
**553**	*Orchis italica*	EtOAc	35.08		-2.05					
**554**	*Orchis italica*	MeOH	22.48		-15.02					
**555**	*Orchis quadripunctata*	EtOAc	29.74		-9.95					
**556**	*Orchis quadripunctata*	MeOH	30.27		-12.44					
**557**	*Origanum dictamnus*	EtOAc	13.00		8.65					
**558**	*Origanum dictamnus*	MeOH	59.22		11.69		4	10.57	>100	87
**559**	*Origanum majorana*	EtOAc	52.50		1.66		20	58.48	69	39
**560**	*Origanum majorana*	MeOH	86.39	52.17	49.27	87.4	100	>100	30	27
**561**	*Origanum microphyllum*	EtOAc	36.99		19.03					
**562**	*Origanum microphyllum*	MeOH	49.00		19.13					

**563**	*Origanum onites*	EtOAc	31.93		-19.00					
**564**	*Origanum onites*	MeOH	45.61		-0.83					
**565**	*Origanum vulgare* subsp. *hirtum*	EtOAc	84.05	47.76	16.61		20	51.5	59	64
**566**	*Origanum vulgare* subsp. *hirtum*	MeOH	85.68	45.52	49.63	89.03	100	>100	57	52
**567**	*Osyris alba*	EtOAc	13.60		13.06					
**568**	*Osyris alba*	MeOH	93.54	28.23	2.66		0.16	1.7	>100	>100
**569**	*Otanthus maritimus*	EtOAc	7.25		6.13					
**570**	*Otanthus maritimus*	MeOH	13.72		14.54					
**571**	*Paeonia mascula* subsp. *hellenica*	EtOAc	25.8		22.3					
**572**	*Paeonia mascula* subsp. *hellenica*	MeOH	95.56	22.47	67.98	33.71	0.16	2.11	>100	>100
**573**	*Pallenis spinosa*	EtOAc	6.89		24.53					
**574**	*Pallenis spinosa*	MeOH	16.79		22.89					
**575**	*Papaver rhoas*	EtOAc	5.43		18.46					
**576**	*Papaver rhoas*	MeOH	17.82		14.07					
**577**	*Parietaria cretica*	EtOAc	10.45		17.08					
**578**	*Parietaria cretica*	MeOH	44.00		16.48					
**579**	*Parietaria diffusa*	EtOAc	4.66		11.70					
**580**	*Parietaria diffusa*	MeOH	40.57		7.19					
**581**	*Paronychia albanica*	EtOAc	32.31		7.93					
**582**	*Paronychia albanica*	MeOH	33.78		6.97					
**583**	*Passiflora incarnata*	EtOAc	54.01		22.78		0.8	1.88	>100	>100
**584**	*Passiflora incarnata*	MeOH	56.34		31.49		20	49.84	86	85
**585**	*Petromarula pinnata*	EtOAc	6.48		20.54					
**586**	*Petromarula pinnata*	MeOH	26.97		14.27					
**587**	*Peucedanum vourinense*	EtOAc	8.27		22.01					

**588**	*Peucedanum vourinense*	MeOH	41.36		8.92				
**589**	*Phlomis cretica*	EtOAc	26.23		4.99				
**590**	*Phlomis cretica*	MeOH	62.04		7.72	4	>100	41	>100
**591**	*Phlomis fruticosa*	EtOAc	18.41		1.55				
**592**	*Phlomis fruticosa*	MeOH	42.32		14.35				
**593**	*Phlomis lanata*	EtOAc	23.54		10.78				
**594**	*Phlomis lanata*	MeOH	74.07		11.97	4	>100	98	>100
**595**	*Phlomis samia*	EtOAc	6.43		18.27				
**596**	*Phlomis samia*	MeOH	32.87		17.67				
**597**	*Phoenix theophrasti*	EtOAc	1.24		-0.92				
**598**	*Phoenix theophrasti*	MeOH	42.91		14.52				
**599**	*Phoenix theophrasti*	EtOAc	7.57		-6.05				
**600**	*Phoenix theophrasti*	MeOH	41.01		5.54				
**601**	*Pimpinella tragium* subsp. *tragium*	EtOAc	11.99		19.88				
**602**	*Pimpinella tragium* subsp. *tragium*	MeOH	29.49		18.95				
**603**	*Pinus heldreichii*	EtOAc	8.72		28.66				
**604**	*Pinus heldreichii*	MeOH	32.84		20.42				
**605**	*Plantago atrata* subsp. *graeca*	EtOAc	7.39		26.04				
**606**	*Plantago atrata* subsp. *graeca*	MeOH	44.64		-1.50				
**607**	*Plantago coronopus* subsp. *cummutata*	EtOAc	7.98		38.09				
**608**	*Plantago coronopus* subsp. *cummutata*	MeOH	29.15		10.41				
**609**	*Plantago holosteum*	EtOAc	46.73		18.94				
**610**	*Plantago holosteum*	MeOH	94.44	26.85	3.08	100	>100	49	38
**611**	*Plantago lanceolata*	EtOAc	30.53		4.72				
**612**	*Plantago lanceolata*	MeOH	49.08		3.74				
**613**	*Plantago major*	EtOAc	39.34		7.61				
**614**	*Plantago major*	MeOH	46.74		9.19				

**615**	*Platanus orientalis*	EtOAc	18.05		-2.40				
**616**	*Platanus orientalis*	MeOH	34.04		-8.80				
**617**	*Polygonum aviculare*	EtOAc	15.54		26.86				
**618**	*Polygonum aviculare*	MeOH	84.93	39.73	23.93	4	>100	94	95
**619**	*Polygonum tinctoria*	EtOAc	17.24		22.84				
**620**	*Polygonum tinctoria*	MeOH	45.34		26.50				
**621**	*Portulaca oleracea*	EtOAc	1.70		12.57				
**622**	*Portulaca oleracea*	MeOH	8.52		13.68				
**623**	*Potentilla speciosa*	EtOAc	24.13		5.18				
**624**	*Potentilla speciosa*	MeOH	94.84	28.44	8.23	100	>100	47	>100
**625**	*Prunella laciniata*	EtOAc	22.04		34.46				
**626**	*Prunella laciniata*	MeOH	94.23	28.12	-0.98	100	>100	56	93
**627**	*Prunus spinosa*	EtOAc	43.51		14.16				
**628**	*Prunus spinosa*	MeOH	42.15		11.61				
**629**	*Pseudorlaya pumila*	EtOAc	11.16		16.23				
**630**	*Pseudorlaya pumila*	MeOH	17.84		13.18				
**631**	*Psoralea bituminosa*	EtOAc	12.12		18.75				
**632**	*Psoralea bituminosa*	MeOH	18.46		-2.12				
**633**	*Pterocephalus perennis* subsp. *perennis*	EtOAc	8.16		20.26				
**634**	*Pterocephalus perennis* subsp. *perennis*	MeOH	39.57		13.72				
**635**	*Ptilostemon afer* subsp. *afer*	EtOAc	10.22		24.95				
**636**	*Ptilostemon afer* subsp. *afer*	MeOH	40.48		17.00				
**637**	*Ptilostemon chamaepeuce*	EtOAc	6.39		28.03				

**638**	*Ptilostemon chamaepeuce*	MeOH	68.94		9.99		20	>100	>100	>100
**639**	*Punica granatum*	EtOAc	48.16		14.30					
**640**	*Punica granatum*	MeOH	94.75	27.54	19.84		100	>100	29	99
**641**	*Putoria calabrica*	EtOAc	12.26		22.53					
**642**	*Putoria calabrica*	MeOH	18.00		7.63					
**643**	*Pyrus spinosa*	EtOAc	16.16		9.88					
**644**	*Pyrus spinosa*	MeOH	84.00	31.19	17.14		100	>100	62	81
**645**	*Quercus ithaburensis* subsp. *macrolepis*	EtOAc	22.72		21.66					
**646**	*Quercus ithaburensis* subsp. *macrolepis*	MeOH	94.41	26.05	27.96		100	>100	27	>100
**647**	*Raphanus sativus*	EtOAc	8.12		10.65					
**648**	*Raphanus sativus*	MeOH	8.89		6.12					
**649**	*Rhamnus alpina*	EtOAc	20.97		21.94					
**650**	*Rhamnus alpina*	MeOH	37.00		-5.36					
**651**	*Rhamnus alpina*	EtOAc	30.06		18.58					
**652**	*Rhamnus alpina*	MeOH	33.49		-0.90					
**653**	*Rhamnus lycioides* subsp. *graeca*	EtOAc	24.10		13.02					
**654**	*Rhamnus lycioides* subsp. *graeca*	MeOH	43.75		2.91					
**655**	*Rhamnus sibthorpiana*	EtOAc	36.81		33.51					
**656**	*Rhamnus sibthorpiana*	MeOH	18.84		5.30					
**657**	*Rosa damascena*	EtOAc	29.66		26.58					
**658**	*Rosa damascena*	MeOH	94.58	27.05	49.85	80.21	0.8	>100	51	61
**659**	*Rosmarinus officinalis*	EtOAc	78.83		18.62		4	12.22	89	>100
**660**	*Rosmarinus officinalis*	MeOH	68.57		10.92		20	48.56	55	100

**661**	*Rubia peregrina*	EtOAc	11.56		38.48					
**662**	*Rubia peregrina*	MeOH	18.71		21.77					
**663**	*Rubia tenuifolia*	EtOAc	9.26		16.84					
**664**	*Rubia tenuifolia*	MeOH	24.64		13.23					
**665**	*Rubia tinctorum*	EtOAc	9.50		10.04					
**666**	*Rubia tinctorum*	MeOH	12.77		9.78					
**667**	*Rubia tinctorum*	EtOAc	30.56		11.26					
**668**	*Rubia tinctorum*	MeOH	16.57		0.53					
**669**	*Ruta graveolens*	EtOAc	10.66		27.64					
**670**	*Ruta graveolens*	MeOH	32.57		22.18					
**671**	*Salvia amplexicaulis*	EtOAc	20.24		25.16					
**672**	*Salvia amplexicaulis*	MeOH	92.91	29.05	7.93		20	>100	87	95
**673**	*Salvia argentea*	EtOAc	11.20		23.46					
**674**	*Salvia argentea*	MeOH	42.31		26.84					
**675**	*Salvia candidissima*	EtOAc	41.09		21.25					
**676**	*Salvia candidissima*	MeOH	39.04		23.39					
**677**	*Salvia fruticosa*	EtOAc	67.92		27.50		4	10.59	89	81
**678**	*Salvia fruticosa*	MeOH	90.68	24.53	44.37	92.68	4	9.95	>100	91
**679**	*Salvia officinalis*	EtOAc	88.69	44.37	23.22		20	52.23	57	66
**680**	*Salvia officinalis*	MeOH	88.01	45.18	49.40	92.14	20	54.23	>100	62
**681**	*Salvia pomifera* subsp. *calycina*	EtOAc	12.22		35.69					
**682**	*Salvia pomifera* subsp. *calycina*	MeOH	89.61	33.31	40.33	88.97	20	60.71	68	82
**683**	*Salvia pomifera* subsp. *pomifera*	EtOAc	57.10		36.46		20	57.33	>100	>100
**684**	*Salvia pomifera* subsp. *pomifera*	MeOH	61.79		15.38		20	5.4	48	>100
**685**	*Salvia ringens*	EtOAc	29.10		34.31					
**686**	*Salvia ringens*	MeOH	93.80	28.46	4.62		20	65.95	78	>100
**687**	*Salvia sclarea*	EtOAc	54.09		23.96		20	42.79	88	69
**688**	*Salvia sclarea*	MeOH	88.17	29.82	45.86	99.64	20	53.24	67	>100
**689**	*Salvia verbenaca*	EtOAc	8.08		24.87					
**690**	*Salvia verbenaca*	MeOH	45.68		16.08					

**691**	*Salvia viridis*	EtOAc	36.94		23.30					
**692**	*Salvia viridis*	MeOH	90.27	22.97	17.32		100	>100	36	>100
**693**	*Sambucus ebulus*	EtOAc	14.94		0.32					
**694**	*Sambucus ebulus*	MeOH	50.72		2.55		100	>100	66	59
**695**	*Sambucus nigra*	EtOAc	3.81		-6.73					
**696**	*Sambucus nigra*	MeOH	14.09		-1.95					
**697**	*Saponaria officinalis*	EtOAc	3.68		18.85					
**698**	*Saponaria officinalis*	MeOH	3.22		10.91					
**699**	*Sarcopoterium spinosum*	EtOAc	17.97		26.52					
**700**	*Sarcopoterium spinosum*	MeOH	43.72		19.18					
**701**	*Satureja hortensis*	EtOAc	84.00	51.19	23.31		100	>100	59	49
**702**	*Satureja hortensis*	MeOH	82.34	43.61	46.82	84.36	100	>100	34	40
**703**	*Satureja horvatii* subsp. *macrophylla*	EtOAc	23.70		-13.25					
**704**	*Satureja horvatii* subsp. *macrophylla*	MeOH	41.37		-4.97					
**705**	*Satureja montana* subsp. *montana*	EtOAc	18.70		18.11					
**706**	*Satureja montana* subsp. *montana*	MeOH	93.14	29.06	11.81		100	>100	64	80
**707**	*Satureja parnassica* subsp. *hellenica*	EtOAc	17.25		31.05					
**708**	*Satureja parnassica* subsp. *hellenica*	MeOH	92.25	20.95	7.24		20	62.9	46	>100
**709**	*Satureja parnassica* subsp. *parnassica*	EtOAc	37.50		5.95					
**710**	*Satureja parnassica* subsp. *parnassica*	MeOH	93.04	19.69	-0.84		100	>100	53	>100
**711**	*Satureja spinosa*	EtOAc	17.86		16.24					
**712**	*Satureja spinosa*	MeOH	93.06	29.06	5.14		20	55.97	93	>100
**713**	*Satureja thymbra*	EtOAc	42.61		-5.27					
**714**	*Satureja thymbra*	MeOH	93.83	28.37	-2.59		20	102.12	74	>100

**715**	*Satureja thymbra (montana ecotype)*	EtOAc	43.01		-14.85					
**716**	*Satureja thymbra (montana ecotype)*	MeOH	92.88	29.81	0.44		100	>100	21	76
**717**	*Scabiosa crenata* subsp. *crenata*	EtOAc	10.56		18.58					
**718**	*Scabiosa crenata* subsp. *crenata*	MeOH	35.88		14.79					
**719**	*Scabiosa crenata* subsp. *crenata*	EtOAc	14.88		34.54					
**720**	*Scabiosa crenata* subsp. *crenata*	MeOH	48.61		22.49					
**721**	*Scandix pecten-veneris*	EtOAc	5.13		20.11					
**722**	*Scandix pecten-veneris*	MeOH	43.81		17.56					
**723**	*Scolymus hispanicus*	EtOAc	5.23		10.96					
**724**	*Scolymus hispanicus*	MeOH	16.95		7.18					
**725**	*Scorzonera crocifolia*	EtOAc	6.34		2.45					
**726**	*Scorzonera crocifolia*	MeOH	10.00		6.98					
**727**	*Scutellaria sieberi*	EtOAc	12.56		4.08					
**728**	*Scutellaria sieberi*	MeOH	11.00		9.85					
**729**	*Sedum album*	EtOAc	6.12		8.76					
**730**	*Sedum album*	MeOH	41.45		9.06					
**731**	*Sedum sediforme*	EtOAc	42.90		-0.39					
**732**	*Sedum sediforme*	MeOH	96.27	22.03	70.55	64.05	100	>100	53	82
**733**	*Selinum seilaifolium*	EtOAc	5.60		28.38					
**734**	*Selinum seilaifolium*	MeOH	8.96		24.50					

**735**	*Senecio eubeus*	EtOAc	5.07	21.44				
**736**	*Senecio eubeus*	MeOH	8.23	13.07				
**737**	*Senecio taygeteus*	EtOAc	21.25	15.33				
**738**	*Senecio taygeteus*	MeOH	37.00	17.07				
**739**	*Senecio thapsoides*	EtOAc	9.39	24.83				
**740**	*Senecio thapsoides*	MeOH	46.20	26.41				
**741**	*Sesamum indicum*	EtOAc	3.65	10.54				
**742**	*Sesamum indicum*	MeOH	12.99	9.99				
**743**	*Seseli rigidum*	EtOAc	3.71	21.73				
**744**	*Seseli rigidum*	MeOH	4.37	29.32				
**745**	*Sideritis clandestina* subsp. *clandestina*	EtOAc	41.66	2.80				
**746**	*Sideritis clandestina* subsp. *clandestina*	MeOH	40.52	12.01				
**747**	*Sideritis clandestina* subsp. *peloponnesiaca*	EtOAc	41.95	6.91				
**748**	*Sideritis clandestina* subsp. *peloponnesiaca*	MeOH	45.23	13.54				
**749**	*Sideritis curvidens*	EtOAc	10.67	-0.21				
**750**	*Sideritis curvidens*	MeOH	18.07	0.07				
**751**	*Sideritis euboea*	EtOAc	33.59	12.56				
**752**	*Sideritis euboea*	MeOH	47.33	6.87				
**753**	*Sideritis perfoliata* subsp. *perfoliata*	EtOAc	48.75	16.89				
**754**	*Sideritis perfoliata* subsp. *perfoliata*	MeOH	50.49	19.52	100	>100	33	26
**755**	*Sideritis raeseri* subsp. *raeseri*	EtOAc	43.41	5.29				
**756**	*Sideritis raeseri* subsp. *raeseri*	MeOH	71.42	9.49	100	>100	100	>100
**757**	*Sideritis romana* subsp. *romana*	EtOAc	20.55	7.15				

**758**	*Sideritis romana* subsp. *romana*	MeOH	37.25		5.28					
**759**	*Sideritis scardica*	EtOAc	91.26	20.27	21.56		20	48.51	88	64
**760**	*Sideritis scardica*	MeOH	92.85	29.24	49.94	84.37	20	51.27	47	64
**761**	*Sideritis syriaca* subsp. *syriaca*	EtOAc	48.59		7.58					
**762**	*Sideritis syriaca* subsp. *syriaca*	MeOH	63.78		4.46		100	>100	39	>100
**763**	*Silene auriculata*	EtOAc	3.18		12.78					
**764**	*Silene auriculata*	MeOH	4.02		14.23					
**765**	*Silene bupleuroides*	EtOAc	8.95		17.39					
**766**	*Silene bupleuroides*	MeOH	14.91		22.07					
**767**	*Silene vulgaris*	EtOAc	4.90		10.37					
**768**	*Silene vulgaris*	MeOH	14.43		19.80					
**769**	*Silybum marianum*	EtOAc	12.49		45.14	45.87				
**770**	*Silybum marianum*	MeOH	56.98		35.94		100	>100	74	50
**771**	*Sinapis alba*	EtOAc	4.16		41.35	83.64				
**772**	*Sinapis alba*	MeOH	8.46		19.17					
**773**	*Solanum melongena*	EtOAc	8.21		14.23					
**774**	*Solanum melongena*	MeOH	39.23		17.50					
**775**	*Solanum melongena*	EtOAc	8.41		17.13					
**776**	*Solanum melongena*	MeOH	36.82		22.46					
**777**	*Sonchus asper*	EtOAc	6.58		24.10					
**778**	*Sonchus asper*	MeOH	21.53		5.53					
**779**	*Sorbus aria*	EtOAc	42.40		19.89					
**780**	*Sorbus aria*	MeOH	93.66	28.77	17.46		20	>100	48	61
**781**	*Sorbus umbellata*	EtOAc	46.11		11.42					
**782**	*Sorbus umbellata*	MeOH	94.81	37.47	22.47		20	77.65	51	>100
**783**	*Stachys chrysantha*	EtOAc	31.26		10.85					
**784**	*Stachys chrysantha*	MeOH	42.50		22.46					
**785**	*Stachys germanica* subsp. *heldreichii*	EtOAc	45.37		13.90					
**786**	*Stachys germanica* subsp. *heldreichii*	MeOH	46.46		20.91					

**787**	*Stachys iva*	EtOAc	14.07		-3.04					
**788**	*Stachys iva*	MeOH	40.93		0.00					
**789**	*Stachys leucoglossa*	EtOAc	15.70		13.63					
**790**	*Stachys leucoglossa*	MeOH	48.95		10.14					
**791**	*Stachys scardica*	EtOAc	28.86		8.30					
**792**	*Stachys scardica*	MeOH	90.69	33.43	14.91		100	>100	64	84
**793**	*Stachys spinosa*	EtOAc	12.33		9.32					
**794**	*Stachys spinosa*	MeOH	25.50		9.07					
**795**	*Stachys spruneri*	EtOAc	24.93		4.72					
**796**	*Stachys spruneri*	MeOH	49.57		3.11					
**797**	*Stachys tymphaea*	EtOAc	40.25		12.26					
**798**	*Stachys tymphaea*	MeOH	90.67	32.21	20.40		100	>100	26	90
**799**	*Staehelina petiolata*	EtOAc	11.11		9.80					
**800**	*Staehelina petiolata*	MeOH	23.06		19.12					
**801**	*Staehelina uniflosculosa*	EtOAc	8.15		7.16					
**802**	*Staehelina uniflosculosa*	MeOH	84.73	50.94	9.83		100	>100	53	86
**803**	*Styrax officinalis*	EtOAc	25.73		17.04					
**804**	*Styrax officinalis*	MeOH	54.22		7.12		20	55.24	>100	>100
**805**	*Tamus communis*	EtOAc	87.57	45.5	-5.58		4	10.17	85	93
**806**	*Tamus communis*	MeOH	59.55		3.04		20	60.2	95	>100
**807**	*Tamus communis*	EtOAc	19.50		35.60					
**808**	*Tamus communis*	MeOH	10.36		25.12					
**809**	*Tanacetum parthenium*	EtOAc	8.91		13.73					
**810**	*Tanacetum parthenium*	MeOH	47.60		16.98					
**811**	*Taxus baccata*	EtOAc	95.48	26.26	13.59		100	>100	100	>100
**812**	*Taxus baccata*	MeOH	94.78	28.62	13.41		0.8	>100	94	>100
**813**	*Taxus baccata*	EtOAc	49.84		-108.54					
**814**	*Taxus baccata*	MeOH	42.06		-23.75					
**815**	*Taxus baccata*	EtOAc	41.86		-72.59					
**816**	*Taxus baccata*	MeOH	46.68		-52.72					

**817**	*Teline monspessulana*	EtOAc	20.47		7.83					
**818**	*Teline monspessulana*	MeOH	26.66		-4.30					
**819**	*Tetragonolobus purpureus*	EtOAc	1.50		27.23					
**820**	*Tetragonolobus purpureus*	MeOH	5.28		18.41					
**821**	*Teucrium capitatum*	EtOAc	28.45		13.85					
**822**	*Teucrium capitatum*	MeOH	88.71	52.73	3.46		100	>100	51	76
**823**	*Teucrium chamaedrys* subsp. *chamaedrys*	EtOAc	48.17		9.62					
**824**	*Teucrium chamaedrys* subsp. *chamaedrys*	MeOH	87.02	46.83	6.55		100	>100	62	>100
**825**	*Teucrium divaricatum* subsp. *divaricatum*	EtOAc	32.86		13.55					
**826**	*Teucrium divaricatum* subsp. *divaricatum*	MeOH	81.85	34.18	9.99		100	>100	56	77
**827**	*Teucrium halacsyanum*	EtOAc	29.96		7.91					
**828**	*Teucrium halacsyanum*	MeOH	42.15		7.50					
**829**	*Teucrium montanum* subsp. *helianthemoiides*	EtOAc	14.98		7.82					
**830**	*Teucrium montanum* subsp. *helianthemoiides*	MeOH	40.40		2.20					
**831**	*Teucrium montanum* subsp.	EtOAc	8.58		-0.22					

	montanum								
**832**	*Teucrium montanum* subsp. *montanum*	MeOH	82.12	43.61	-0.99	20	>100	48	>100
**833**	*Thalictrum minus* subsp. *olympicum*	EtOAc	13.77		18.08				
**834**	*Thalictrum minus* subsp. *olympicum*	MeOH	47.93		12.42				
**835**	*Thapsia garganica*	EtOAc	4.65		18.96				
**836**	*Thapsia garganica*	MeOH	20.66		16.84				
**837**	*Thymelaea hirsuta*	EtOAc	23.59		14.44				
**838**	*Thymelaea hirsuta*	MeOH	43.26		10.96				
**839**	*Thymelaea tartonraira* subsp. *argentea*	EtOAc	18.61		9.65				
**840**	*Thymelaea tartonraira* subsp. *argentea*	MeOH	47.61		13.94				
**841**	*Thymus atticus*	EtOAc	39.63		4.56				
**842**	*Thymus atticus*	MeOH	48.67		2.46				
**843**	*Thymus boissieri* var. *boissieri*	EtOAc	6.03		30.63				
**844**	*Thymus boissieri* var. *boissieri*	MeOH	44.30		12.22				
**845**	*Thymus leucospermus*	EtOAc	43.12		-14.18				
**846**	*Thymus leucospermus*	MeOH	92.26	30.79	-2.97	100	>100	28	>100
**847**	*Thymus leucotrichus*	EtOAc	13.94		38.35				
**848**	*Thymus leucotrichus*	MeOH	90.53	32.45	8.34	20	>100	44	>100
**849**	*Thymus longicaulis*	EtOAc	32.16		2.94				
**850**	*Thymus longicaulis*	MeOH	92.29	20.23	7.20	100	>100	64	50

**851**	*Thymus vulgaris*	EtOAc	86.84	32.14	23.12		20	55.89	78	44
**852**	*Thymus vulgaris*	MeOH	86.31	33.04	44.93	71.02	100	>100	57	45
**853**	*Tragopogon porrifolius*	EtOAc	6.29		12.50					
**854**	*Tragopogon porrifolius*	MeOH	23.25		15.06					
**855**	*Trifolium noricum*	EtOAc	8.48		11.11					
**856**	*Trifolium noricum*	MeOH	13.23		1.01					
**857**	*Trifolium noricum*	EtOAc	6.80		19.58					
**858**	*Trifolium noricum*	MeOH	11.23		16.49					
**859**	*Trifolium pratense*	EtOAc	12.25		21.46					
**860**	*Trifolium pratense*	MeOH	40.41		7.33					
**861**	*Tussilago farfara*	EtOAc	17.60		22.53					
**862**	*Tussilago farfara*	MeOH	80.32	56.14	23.75		100	>100	42	83
**863**	*Umbilicus horizontalis*	EtOAc	18.77		18.18					
**864**	*Umbilicus horizontalis*	MeOH	94.89	27.03	80.95	69.08	100	>100	51	95
**865**	*Urtica pilulifera*	EtOAc	3.69		14.04					
**866**	*Urtica pilulifera*	MeOH	10.37		18.20					
**867**	*Valeriana italica*	EtOAc	27.86		1.59					
**868**	*Valeriana italica*	MeOH	35.21		3.71					
**869**	*Valeriana italica*	EtOAc	17.34		7.67					
**870**	*Valeriana italica*	MeOH	34.26		11.87					
**871**	*Valeriana tuberosa*	EtOAc	15.83		8.81					
**872**	*Valeriana tuberosa*	MeOH	21.16		16.74					
**873**	*Veratrum album*	EtOAc	17.30		59.07	96.31				
**874**	*Veratrum album*	MeOH	23.60		77.64	74.58				
**875**	*Verbascum acaule*	EtOAc	10.59		14.46					
**876**	*Verbascum acaule*	MeOH	17.27		13.69					
**877**	*Verbascum arcturus*	EtOAc	28.89		1.89					
**878**	*Verbascum arcturus*	MeOH	58.33		-3.14		20	52.47	100	>100
**879**	*Verbascum arcturus*	EtOAc	38.97		-0.63					
**880**	*Verbascum arcturus*	MeOH	56.95		-0.47		20	>100	>100	>100

**881**	*Verbascum daenzeri*	EtOAc	18.55	13.10
**882**	*Verbascum daenzeri*	MeOH	22.46	2.92
**883**	*Verbascum epixanthinum* var. *epixanthinum*	EtOAc	41.84	2.28
**884**	*Verbascum epixanthinum* var. *epixanthinum*	MeOH	45.33	5.48
**885**	*Verbascum macrurum*	EtOAc	45.17	3.21
**886**	*Verbascum macrurum*	MeOH	47.38	10.40
**887**	*Verbascum undulatum*	EtOAc	32.41	1.60
**888**	*Verbascum undulatum*	MeOH	45.46	-3.06
**889**	*Verbena officinalis*	EtOAc	17.22	2.35
**890**	*Verbena officinalis*	MeOH	35.62	-2.27
**891**	*Vicia faba*	EtOAc	9.77	36.58
**892**	*Vicia faba*	MeOH	43.00	24.65
**893**	*Vincetoxicum creticum*	EtOAc	13.32	20.94
**894**	*Vincetoxicum creticum*	MeOH	26.19	23.64
**895**	*Viscum album (on Abies cephalonica)*	EtOAc	10.06	18.11
**896**	*Viscum album (on Abies cephalonica)*	MeOH	22.39	15.03
**897**	*Vitex agnus-castus*	EtOAc	5.34	2.18
**898**	*Vitex agnus-castus*	MeOH	11.71	-2.79
**899**	*Vitex agnus-castus*	EtOAc	27.62	16.85
**900**	*Vitex agnus-castus*	MeOH	46.80	12.99
